# Elevated Levels of Ultrashort- and Short-Chain Perfluoroalkyl Acids in US Homes and People

**DOI:** 10.1021/acs.est.2c06715

**Published:** 2023-10-11

**Authors:** Guomao Zheng, Stephanie M. Eick, Amina Salamova

**Affiliations:** † School of Environmental Science and Engineering, Southern University of Science and Technology, Shenzhen 518055, China; ‡ Gangarosa Department of Environmental Health, Rollins School of Public Health, Emory University, Atlanta, Georgia 30322, United States; § Department of Epidemiology, Rollins School of Public Health, Emory University, Atlanta, Georgia 30322, United States

**Keywords:** PFAS, PFAAs, trifluoroacetic acid (TFA), perfluoropropanoic acid (PFPrA), ultrashort-chain PFAS, short-chain PFAS, PFAA precursors, indoor exposure, biomonitoring

## Abstract

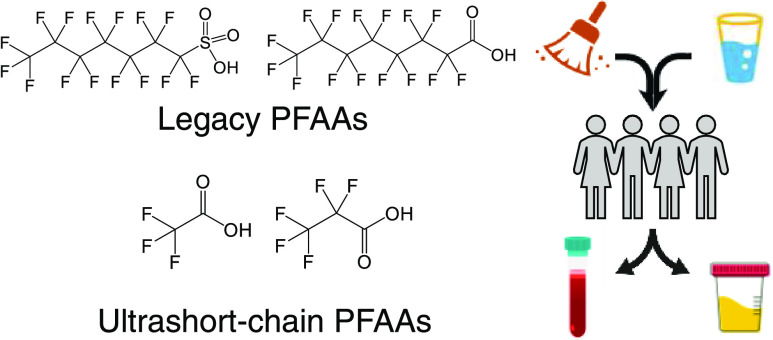

Per- and polyfluoroalkyl substances (PFAS) make up a large group of fluorinated organic compounds extensively used in consumer products and industrial applications. Perfluorooctanesulfonic acid (PFOS) and perfluorooctanoic acid (PFOA), the two perfluoroalkyl acids (PFAAs) with 8 carbons in their structure, have been phased out on a global scale because of their high environmental persistence and toxicity. As a result, shorter-chain PFAAs with less than 8 carbons in their structure are being used as their replacements and are now widely detected in the environment, raising concerns about their effects on human health. In this study, 47 PFAAs and their precursors were measured in paired samples of dust and drinking water collected from residential homes in Indiana, United States, and in blood and urine samples collected from the residents of these homes. Ultrashort- (with 2 or 3 carbons [C2–C3]) and short-chain (with 4–7 carbons [C4–C7]) PFAAs were the most abundant in all four matrices and constituted on average 69–100% of the total PFAA concentrations. Specifically, trifluoroacetic acid (TFA, C2) and perfluoropropanoic acid (PFPrA, C3) were the predominant PFAAs in most of the samples. Significant positive correlations (*n* = 81; *r* = 0.23–0.42; *p* < 0.05) were found between TFA, perfluorobutanoic acid (PFBA, C4), and perfluoroheptanoic acid (PFHpA, C7) concentrations in dust or water and those in serum, suggesting dust ingestion and/or drinking water consumption as important exposure pathways for these compounds. This study demonstrates that ultrashort- and short-chain PFAAs are now abundant in the indoor environment and in humans and warrants further research on potential adverse health effects of these exposures.

## Abstract 2

This study demonstrates that ultrashort- and short-chain PFAAs are now prevalent in the indoor environment and in humans.

## Introduction

Per- and polyfluoroalkyl substances (PFAS) are a large group of fluorinated organic compounds that are extensively used in various industrial and consumer applications such as water-, grease-, and stain-repellents, surfactants, and lubricants.^1^ Perfluorooctanoic acid (PFOA) and perfluorooctanesulfonic acid (PFOS), commonly referred to as legacy PFAS, are perfluoroalkyl acids (PFAAs) with 8 carbons in their structure (C8) that have been widely used since the 1940s. However, their manufacturing and use have been regulated over the last two decades due to their environmental persistence, bioaccumulation, and toxicity to wildlife and humans.^2^ PFOS was added to the Annex B (global restriction) of the Stockholm Convention on Persistent Organic Pollutants in 2009 and PFOA was listed under Annex A (global elimination) of the convention in 2019.^3^ As a result of these restrictions, other PFAS have become more widely used, including shorter-chain PFAAs (with less than 8 carbons in their structure) that were introduced as less persistent and bioaccumulative alternatives because of their smaller molecular size.^4^ However, recent studies have shown that short-chain PFAAs are now ubiquitous in indoor and outdoor environments. Several of these shorter-chain compounds have been detected in indoor dust,^5^ air,^6^ drinking water,^7,8^ aquatic systems,^9^ soil, and sediment.^10,11^ It also has been shown that short-chain PFAAs are mobile^12^ and can be transported over long distances through seawater and reach remote areas.^13,14^ Moreover, the two ultrashort-chain PFAAs, trifluoroacetic acid (TFA, C2) and perfluoropropanoic acid (PFPrA, C3), have also been found in the environment, including snow, surface, and groundwater,^7,13^ precipitation,^15,16^ sediment, soils, and sludge.^17^ TFA and PFPrA are industrial chemicals, used as laboratory reagents and catalysts, as well as byproducts in chemical synthesis.^18,19^ In addition, these two compounds can be formed from various indirect sources, including atmospheric oxidation of PFAA precursors or chlorofluorocarbons,^1,20,21^ novel refrigerants,^13,22^ the photodegradation or thermolysis of fluoropolymers in the environment,^23^ and biological degradation of plant-protecting agents or pharmaceuticals,^7,15,20^ all posing as potential sources of the ultrashort-chain PFAAs in the environment. Furthermore, multiple studies suggest that PFAA precursors, such as side-chain fluorotelomer-based polymers, fluorotelomer alcohols (FTOHs), and polyfluoroalkyl phosphate esters (PAPs), as well as long-chain PFAAs (with more than 8 carbons in their structure), can degrade to shorter-chain PFAAs in the environment.^9,23,24^ Consequently, due to their high persistence and mobility,^25,26^ perfluorobutanesulfonic acid (PFBS, C4) and its salts were identified as substances of very high concern under the European Union Registration, Evaluation, Authorization, and Restriction of Chemicals (REACH) Program in 2019,^27^ and concerns were also raised calling for the urgent and effective regulation of other short-chain PFAAs.^28^

In recent years, the ultrashort- and short-chain PFAAs have also been detected in human blood,^29−31^ breast milk,^32,33^ and urine.^34^ A study from Tianjin, China, reported that TFA was commonly detected at levels comparable to those of several long-chain PFAAs in human blood.^18^ The levels of PFBS in Swedish women’s blood increased at a rate of around 11% per year between 1996 and 2010.^31^ The detection frequencies of several short-chain PFAAs (C4–C7) have been increasing in breast milk with a doubling time of ∼4 years on a global scale from 1996 to 2019.^32^ Perfluorobutanoic acid (PFBA, C4), perfluorohexanoic acid (PFHxA, C6), and perfluoroheptanoic acid (PFHpA, C7) were frequently detected in urine samples taken from the general population in the United States in 2013–2014.^34^

There is growing evidence of the toxicity of some ultrashort- and short-chain PFAAs. The acute toxicity of TFA and PFPrA on freshwater invertebrates was found to be higher than that of the longer-chain PFAAs.^35^*In vitro* and *in vivo* studies have demonstrated that exposure to short-chain PFAAs, such as PFBA, PFBS, PFHxA, and PFHpA, can have adverse effects on the reproductive, developmental, hepatic, and renal systems as well as lipid metabolism.^36−43^ Recent epidemiological research demonstrates that PFBS and PFHpA can disrupt gonadotropins as well as free androgen levels in fetuses.^44^ Moreover, perfluoropentanoic acid (PFPeA, C5) can alter the thyroid function in newborns, further indicating the developmental toxicity of these short-chain PFAAs in humans.^45^

There are several pathways through which humans can be exposed to ultrashort- and short-chain PFAAs. PFAAs with a shorter carbon chain were more frequently detected in U.S. bottled water compared to the long-chain PFAAs, with PFPrA contributing 42% to the detected PFAA concentrations.^46^ In addition, PFBA, PFBS, PFPeA, PFHxA, and PFHpA were frequently detected in source and treated drinking water collected from 25 water treatment plants in the U.S. (detection frequencies [DF]: 88–100%).^8^ A recent study from China has also found ultrashort- and short-chain PFAAs in indoor and outdoor dust, with TFA as the most abundant.^5^ Moreover, our previous study has demonstrated that breastfeeding is an important exposure pathway to short-chain PFAAs (C4–C7) in nursing infants.^32^ In addition, biotransformation of PFAA precursors can contribute to human exposure to short-chain PFAAs.^47−50^ For example, biotransformation of perfluorophosphate monoesters (monoPAPs), PAP diesters (diPAPs), and polyfluoroalkyl carboxamides has resulted in the formation of shorter-chain PFAAs in *in vivo* studies.^49,51^ Multiple studies have indicated that the precursors of short-chain PFAAs are ubiquitous in indoor dust,^5,52−54^ air,^55,56^ and consumer products (*e.g.*, cosmetics and food packaging materials);^57−59^ however, the significance of their biotransformation to shorter-chain PFAAs as an indirect exposure pathway in humans remains largely unknown.

In this study, we analyzed paired samples of residential dust and drinking water collected from households in Indiana, United States, as well as paired blood serum and urine samples collected from the residents of these homes (total *n* = 324 of matched serum, urine, dust, and drinking water samples collected from 81 participants) for a suite of 47 PFAAs and their precursors. These included 2 ultrashort- (2–3 carbons) and 4 short-chain (4–7 carbons) perfluorocarboxylic acids (PFCAs), 1 ultrashort- (3 carbons) and 2 short-chain (4 or 5 carbons) perfluoroalkanesulfonic acids (PFSAs), and 14 long-chain PFAAs [PFCAs with >7 carbons and PFSAs with >5 carbons], as well as 24 PFAA precursors (fluorotelomer sulfonates [FTSAs], perfluorooctane sulfonamides/perfluorooctane sulfonamidoethanols [FOSAs/FOSEs], polyfluorinated phosphate esters [PAPs], fluorotelomer alcohols [FTOHs], and fluorotelomer acrylates/fluorotelomer methacrylates [FTACs/FTMACs]). Our goal was to examine the current PFAS exposure patterns in people and their residences, investigate the associations between the chemical levels in four sampled matrices, and estimate the relative contributions of dust and drinking water uptake to the overall body burden.

## Materials and Methods

### Sample Collection

All samples, including dust, drinking water, blood, and urine, were collected between the months of August and December 2020 in the state of Indiana, United States. Participants (*n* = 81) were recruited from the Person to Person (P2P) Health Interview Study cohort (https://precisionhealth.iu.edu/get-involved/person-to-person.html). The study was approved by the Indiana University Institutional Review Board and all participants signed an informed consent form before participation. Dust, drinking water, blood, and urine were all paired and collected on the same day (one dust, water, blood, and urine sample per participant and their household; total *n* = 324; 4 samples per participant). All samples were kept in a cooler with ice packs before being delivered to the laboratory at the end of each sampling day. Blood serum separation was conducted on the day the samples were delivered to the laboratory. The samples were stored at −80 °C until analysis. Demographic, behavioral, and housing information was collected from each participant using questionnaires administered at the time of sample collection (see Table S1 for detailed survey questions).

### Sample Analysis

All samples were analyzed for 47 PFAS, including 23 PFAAs (14 PFCAs and 9 PFSAs) and 24 PFAA precursors (3 FTSAs, 7 FOSAs/FOSEs, 5 PAPs, 4 FTOHs, and 5 FTACs/FTMACs) using liquid chromatography- and gas chromatography–mass spectrometry. The complete list of analytes and the details of the analytical methods and quality control measures (method validation results, method detection limits [MDLs], blank levels, and surrogate and matrix spike recoveries) are provided in the Supporting Information (Tables S2–S6).

### Data Analysis

The distribution of demographic and housing characteristics in the study population was examined using means (with their standard deviations), frequencies, and counts. Separately for each matrix, detection frequencies for all PFAAs and PFAA precursors were assessed, and selected percentiles (minimum, median, maximum) were used to examine distributions of analyte concentrations. The total concentrations were defined as the sum of all of the analyte concentrations measured for that specific analyte group. The contribution of each analyte to the total concentration was determined based on the ratio of the median concentration of that analyte to the median total concentration.

For the statistical analyses, concentrations below method detection limits (MDLs) were imputed with MDL/2. The reported concentrations were blank corrected by subtracting the average blank levels from the sample levels. All PFAA and PFAA precursor concentrations were logarithmically transformed (natural log) for downstream analyses. Correlations between the concentrations of PFAAs and PFAA precursors detected in more than 50% of the samples across matrices were examined by using Spearman correlation coefficients.

A one-compartment toxicokinetic (TK) model^60^ was applied to estimate the resulting serum concentrations (*C*_dust to serum_ and *C*_water to serum_) and relative source contributions (RSCs) of dust (dust ingestion + dust dermal absorption) and drinking water intake (RSC_dust to serum_ and RSC_water to serum_) for PFAAs with >50% detection. The details of this analysis are presented in the Supporting Information. Lastly, we assessed whether concentrations of total PFAA and PFAA precursor varied across housing characteristics using a Mann–Whitney test for the comparison of the logarithmically transformed concentrations.

All statistical analyses were conducted using IBM SPSS Statistics 24 and Sigma Plot 13.

## Results and Discussion

### Population Characteristics

A summary of the participants’ demographic and housing characteristics (*n* = 81) is given in Table 1. Participants ranged in age from 25 to 88 years old (mean 49 ± 16 years), with 36% males and 64% females. Twenty-seven percent of participants had attained a college education and 35% had some college experience. Thirty-three percent were smokers. Twenty-eight percent of participants had a BMI within the normal range (18.5–24.9 kg/m^2^), while 67% were overweight (25–29.9 kg/m^2^) or obese (≥30 kg/m^2^). Participants spent, on average, about 17 ± 5.0 h per day in their homes. None of the participants worked in an environment posing occupational PFAS exposure (*e.g.*, fire stations).

**Table 1:** Summary of Participants’ (*n* = 81) Demographic and Housing Characteristics

demographic characteristics	average (±SD^a^)	*N*	percentage, %	housing characteristics	*N*	percentage, %
age (years)	49 ± 16			water source		
sex				tap	73	90
male		29	36	well	8	10
female		52	64	flooring type		
education				carpet	66	82
high school or less		31	38	other	14	17
some college		28	35	missing	1	1
college or higher		22	27	property type		
smoking				house	54	67
smoker		27	33	apartment	18	22
nonsmoker		54	67	mobile home or other	9	11
BMI (kg/m^2^)				residence built		
underweight, <18.5		4	5	less than 5 years	11	14
normal,18.5–24.9		23	28	5–10 years	20	25
overweight, 25–29.9		13	16	11–20 years	25	31
obese, >30		41	51	31–40 years	10	12
time at home (h)	17 ± 5			missing	15	18
				wallpaper		
				vinyl	62	77
				nonvinyl	12	15
				missing	7	8
				vacuuming frequency		
				never or some days	43	53
				most days/every day	36	44
				missing	2	3

a SD: Standard deviation.

Tap water was the major drinking water source for 90% of the households, while 10% used private wells. Eighty-two percent of homes had some carpet coverage, while the rest had other flooring types (*e.g.*, vinyl or hardwood), and 77% reported having vinyl wallpaper in their homes. Seventy percent of homes were less than 20 years old. More than half of the homes were vacuumed less frequently (some days), while 44% were vacuumed most days or every day (Table 1).

### Concentrations

Overall, of the 47 targeted PFAS, 39 were detected in the analyzed samples. The rest of the analytes were not detected in any of the samples and are not discussed further. The distribution of the detected 39 PFAS, which included 3 ultrashort-, 6 short-, and 14 long-chain PFAAs and 16 PFAA precursors, is presented in Table 2.

**Table 2:** Detection Frequencies (DF, %), Median (Med), Minimum (Min), and Maximum (Max) Concentrations of the Ultrashort-, Short-, and Long-Chain PFAAs and PFAA Precursors Detected in Paired Dust (ng/g), Drinking Water (ng/L), Serum (ng/mL), and Urine^a^ Samples (ng/mL), and Contribution (Contr., %) of Each PFAA or Precursor to the Total Concentrations

	dust			drinking water			serum			urine^a^		
	DF	Med (Min-Max)	Contr.	DF	Med (Min-Max)	Contr.	DF	Med (Min-Max)	Contr.	DF	Med (Min-Max)	Contr.
Ultrashort-Chain												
TFA (C2)	84	220 (ND,^b^ 1400)	75	95	79 (ND, 210)	84	74	6.0 (ND, 77)	57	31	ND (ND, 290)	
PFPrA (C3)	99	26 (ND, 200)	9.1	95	6.9 (ND, 19)	7.4	99	1.0 (0.14, 2.9)	9.5	56	0.051 (ND, 6.8)	1.4
PFPrS (C3)	3.7	ND (ND, 53)		64	0.10 (ND, 0.40)	0.11	4.9	ND (ND, 0.013)		1.2	ND (ND, 0.85)	
**∑ ultrashort-chain**	**100**	**290 (37, 1400)**	**85**	**100**	**86 (9.3, 220)**	**92**	**100**	**6.9 (2.3, 78)**	**66**	**100**	**0.13 (0.02, 290)**	**2.0**
Short-Chain												
PFBA (C4)	94	14 (ND, 410)	5.0	98	2.4 (ND, 7.8)	2.6	84	0.19 (ND, 2.5)	1.8	60	0.33 (ND, 26)	9.2
PFBS (C4)	54	0.40 (ND, 210)	0.14	86	1.3 (ND, 0.16)	1.4	85	0.05 (ND, 0.38)	0.47	3.7	ND (ND, 0.028)	
PFPeA (C5)	10	ND (ND, 120)		70	2.5 (ND, 7.7)	2.6	25	ND (ND, 2.2)		88	3.2 (ND, 34)	89
PFPeS (C5)	22	ND (ND, 15)		59	0.035 (ND, 22)	0.038	69	0.0076 (ND, 0.034)	0.071	23	ND (ND, 0.022)	
PFHxA (C6)	89	4.3 (ND, 290)	1.5	85	0.42 (ND, 6.1)	0.45	83	0.034 (ND, 0.10)	0.32	2.5	ND (ND, 0.09)	
PFHpA (C7)	81	1.7 (ND, 460)	0.60	83	0.15 (ND, 1.2)	0.16	79	0.016 (ND, 0.10)	0.15	23	ND (ND, 0.0093)	
**∑ short-chain**	**100**	**27 (1.4, 1100)**	**7.3**	**100**	**8.5 (0.12, 38)**	**7.2**	**100**	**0.41 (0.058, 3.6)**	**3.0**	**100**	**4.6 (0.021, 41)**	**98**
Long-Chain												
PFHxS (C6)	73	2.7 (ND, 2200)	0.93	88	0.17 (ND, 1.1)	0.18	99	0.78 (ND, 5.4)	7.3	0		
PFHpS (C7)	23	ND (ND, 12)		15	ND (ND, 0.071)		96	0.099 (ND, 0.73)	0.93	0		
PFOA (C8)	98	5.9 (ND, 1900)	2.1	93	0.46 (ND, 3.6)	0.49	99	0.63 (ND, 4.9)	5.9	14	ND (ND, 0.051)	
PFOS (C8)	95	10 (ND, 1100)	3.5	84	0.22 (ND, 1.6)	0.23	99	1.5 (ND, 33)	14	7.4	ND (ND, 0.019)	
PFECHS (C8)	2.5	ND (ND, 7.5)		44	ND (ND, 0.67)		85	0.011 (ND, 0.079)	0.11	0		
PFNA (C9)	64	0.65 (ND, 27)	0.23	65	0.11 (ND, 0.47)	0.11	98	0.21 (ND, 1.2)	2.0	30	ND (ND, 8.9)	
PFNS (C9)	7.4	ND (ND, 1.4)		2.5	ND (ND, 0.015)		1.2	ND (ND, 0.0031)		0		
PFDA (C10)	70	1.8 (ND, 39)	0.62	49	ND (ND, 0.28)		93	0.051 (ND, 0.21)	0.48	0		
PFDS (C10)	36	ND (ND, 100)		0			7.4	ND (ND, 0.019)		0		
PFUdA (C11)	58	0.30 (ND, 15)	0.11	15	ND (ND, 0.093)		79	0.037 (ND, 0.16)	0.35	0		
PFDoA (C12)	70	1.1 (ND, 22)	0.38	25	ND (ND, 0.14)		42	ND (ND, 0.034)		0		
PFTrDA (C13)	57	0.25 (ND, 16)	0.089	14	ND (ND, 0.13)		37	ND (ND, 0.079)		0		
PFTeDA (C14)	58	0.52 (ND, 13)	0.18	26	ND (ND, 0.21)		36	ND (ND, 0.043)		0		
PFHxDA (C16)	42	ND (ND, 8.6)		44	ND (ND, 1.0)		70	0.023 (ND, 0.13)	0.21	0		
**∑ long-chain**	**100**	**33 (0.45, 3300)**	**8.1**	**98**	**1.4 (ND, 5.8)**	**1.0**	**99**	**3.8 (ND, 35)**	**31**	**49**	**ND (ND, 8.9)**	
**∑ PFAAs**	**100**	**360 (11, 4000)**	**100**	**100**	**100 (12, 250)**	**100**	**100**	**13 (3.5, 81)**	**100**	**100**	**8.0 (0.041, 300)**	**100**
PFAA Precursors												
4:2 FTSA	12	ND (ND, 40)		0			0			0		
6:2 FTSA	77	2.6 (ND, 870)	0.78	26	ND (ND, 0.57)		11	ND (ND, 0.62)		7	ND (ND, 0.45)	
8:2 FTSA	64	0.82 (ND, 490)	0.25	0			11	ND (ND, 0.11)		0		
6:2 PAP	78	130 (ND, 5400)	39	0			0			0		
8:2 PAP	51	1.1 (ND, 940)	0.32	0			0			0		
6:2 diPAP	100	120 (2.4, 1900)	37	0			0			0		
6:2/8:2 diPAP	100	20 (0.36, 1900)	6.1	0			0			0		
8:2 diPAP	100	2.9 (0.029, 590)	0.86	0			0			0		
6:2 FTOH^c^	35	ND (ND, 44000)		0			0			0		
8:2 FTOH^c^	64	52 (ND, 33000)	16	0			0			0		
10:2 FTOH^c^	42	ND (ND, 25000)		0			0			0		
8:2 FTMAcr^c^	6.2	ND (ND, 5.4)		0			0			0		
FBSA	0			31	ND (ND, 1.0)		0			10	ND (ND, 0.041)	
FOSA	27	ND (ND, 87)		11	ND (ND, 0.10)		44	ND (ND, 0.36)		0		
MeFOSE^c^	48	ND (ND, 19000)		0			0			0		
EtFOSE^c^	46	ND (ND, 100000)		0			0			0		
**∑ PFAA precursors**	**100**	**850 (23, 110000)**	**100**	**46**	**ND (ND, 1.0)**		**54**	**0.052 (ND, 0.64)**		**15**	**ND (ND, 0.45)**	

a Urine concentrations were adjusted for specific gravity.

b ND: nondetect.

c These PFAA precursors were not determined in water, serum, and urine because of the high volatility of these compounds.

Overall, the ultrashort- and short-chain PFAAs were the predominant PFAAs in all of the samples. Specifically, the ultrashort C2 and C3 PFAAs were the most abundant PFAAs in dust, drinking water, and serum, while the short-chain C4 and C5 PFAAs were predominant in urine. These ultrashort- and short-chain PFAAs contributed 69–100% to the total PFAA concentrations. The precursors were detected primarily in dust, with a few detections in other matrices.

#### Dust

**Figure 1:**
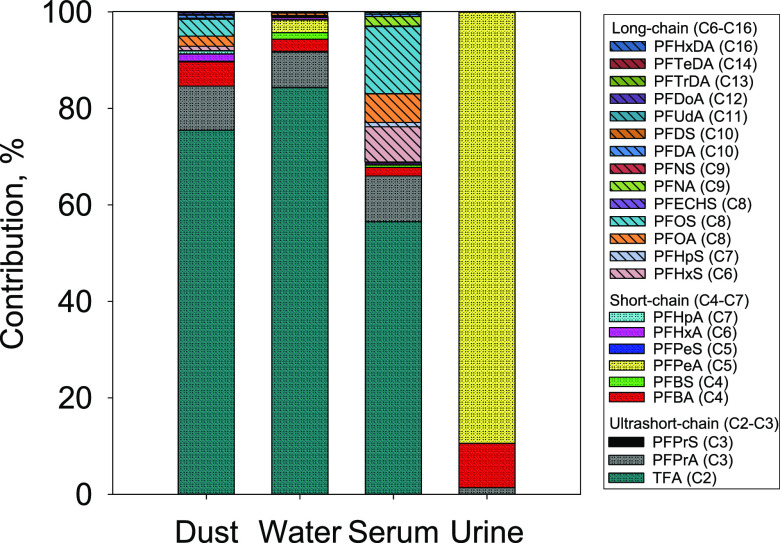
Percent contributions (%; calculated based on median concentrations) of individual ultrashort-, short-, and long-chain PFAAs to the total PFAA concentrations in dust, water, serum, and urine.

TFA, a PFAA with the shortest carbon chain, was detected in 84% of the dust samples and was by far the most abundant PFAA (median of 220 ng/g), contributing 75% to the dust total PFAA concentration (the sum of all detected PFAA concentrations, Figure 1 and Table 2). Another ultrashort-chain PFAA, PFPrA (C3), was the second most abundant PFAA in dust (median 26 ng/g), detected in 99% of the samples, and contributed 9.1% to the dust total PFAA concentration. Among the C4–C7 PFAAs, PFBA (C4), PFHxA (C6), and PFHpA (C7) were the most frequently detected (81–94%) but found at lower concentrations (medians 1.7–14 ng/g) compared to the ultrashort-chain PFAAs, contributing only 0.60–5.0% to the total PFAA concentration in dust.

These findings were similar to those from a recent study from China that reported TFA and PFPrA as the predominant PFAAs in indoor dust (sampling year 2017)^5^ with concentrations of 116–470 ng/g and 35–152 ng/g, respectively, which were similar to the concentrations found in our samples. The levels and detection frequencies of the other short-chain PFAAs detected in dust were lower than those reported in the latter study (range 0.53–152 ng/g; detection frequency [DF] 56–100%).^5^

The two C8 PFAAs, PFOA and PFOS, were also frequently detected in dust (98 and 95%, respectively), but their concentrations (medians of 5.9 and 10 ng/g, respectively) were lower than those of the ultrashort-chain PFAAs. These concentrations were similar to those measured in dust collected from North Carolina in 2014–2016 (7.9 and 4.4 ng/g for PFOA and PFOS, respectively),^61^ but were up to 7 times lower than those found in dust collected in earlier years, such as from Massachusetts in 2009 (24 and 27 ng/g, respectively)^62^ and Wisconsin in 2008 (44 and 47 ng/g, respectively).^63^

Among other long-chain PFAAs, perfluorohexanesulfonic acid (PFHxS) was found at a median concentration of 2.7 ng/g (DF 73%), which was lower than that for PFOS and PFOA. The rest of the long-chain PFAAs, with a few exceptions, were frequently detected (up to 70%) but contributed only a minor portion to the total PFAA concentrations (<1%).

PFAA precursors were primarily detected in dust, with mono- and diPAPs found in 51–100% of the samples (Table 2). The concentrations of precursors in dust reached up to 110,000 ng/g, with 6:2 PAP and 6:2 diPAP detected at the highest levels (medians 130 and 120 ng/g, respectively). The concentrations of 6:2 PAP were higher than those in household dust collected in Stockholm, Sweden (sampling year: 2013–2014; median 31 ng/g), but the levels of 6:2 diPAP were comparable to those found in residential homes in the United States (sampling years: 2014–2016; median 113 ng/g).^61^ In addition, 8:2 FTOH was detected in 64% of the samples at a median concentration of 52 ng/g. Other precursors were detected less frequently and at lower concentrations, except for 2-(*N*-ethylperfluorooctanesulfonamido)-ethanol (EtFOSE) that was found at concentrations reaching up to 100,000 ng/g. Exceptionally high concentrations of EtFOSE reaching up to 75,500 ng/g reported previously^53,55,64^ have been associated with the extensive use of carpets or fabric treatments in homes.^55,65^ The median total precursor concentration in dust (the sum of the 16 detected precursors) was 850 ng/g, twice as high as the median dust total PFAA concentration (360 ng/g), even though the total precursor concentrations were likely underestimated because only several precursors were measured.^54^

#### Drinking Water

TFA was the predominant PFAA in drinking water (median of 79 ng/L) with a detection frequency of 95% and an 84% contribution to the total PFAA concentration in drinking water. PFPrA was the second most abundant PFAA in drinking water (median 6.9 ng/L) detected in 95% of the samples, contributing 7.4% to the total PFAA concentration. The short-chain PFAAs were frequently detected as well but at lower concentrations than TFA and PFPrA and contributed up to 2.6% to the total PFAA concentration. Generally, the detection frequency for the ultrashort- and short-chain PFAAs was higher in drinking water compared to dust. For example, PFPeA was detected in 70% of drinking water samples but only in 10% of dust samples. Similarly, perfluoropropanesulfonic acid (PFPrS, C3), PFBS (C4), and perfluoropentanesulfonic acid (PFPeS, C5) were frequently found in drinking water (64, 86, and 59%, respectively) but less so in dust (3.7, 54, and 22%, respectively).

The levels of TFA in drinking water found in our study were consistent with those detected in drinking water from the United States collected in 1994–1995 (range 41–150 ng/L),^66^ but lower than those reported from China in 2012 (median 155 ng/L).^67^ The levels of the short-chain PFAAs detected in this study (0.035–2.5 ng/L) were comparable to those collected from the 24 states across the United States (0.79–3.6 ng/L).^8^ Inefficient water filtration could be the cause of the frequent detection of short-chain PFAAs in tap water because of the challenges in the removal of these compounds by wastewater treatment processes^7,12^ or by drinking water filters.^68^

A total oxidizable precursor (TOP) assay showed that unknown precursors of TFA and PFPrA constituted up to 94% of the total measured PFAA concentrations in biochemically treated leachate.^69^ Abiotic transformation studies of 6:2 diPAP or of long-chain PFAAs (*e.g.*, PFOA and PFOS) have also reported that their oxidation can lead to the formation of shorter-chain PFAAs.^70,71^ In addition, fluorinated gases such as hydrochlorofluorocarbons and hydrofluorocarbons, fluorinated pesticides and pharmaceuticals, some plastics (*e.g.*, polytetrafluoroethylene), and aqueous film-forming foams have been identified as precursors of TFA in the environment^72^ and may at least partially explain the abundance of TFA in dust and drinking water found in the current study.

PFOA and PFOS were also frequently detected in drinking water (93 and 84%, respectively), but their concentrations were lower than those of the shorter-chain PFAAs. The levels of PFOA and PFOS in drinking water measured here (medians of 0.46 and 0.22 ng/L, respectively) were 2–10 times lower than those in the nationwide studies from the United States in 1989 (0.96 and 1.6 ng/L, respectively) and in 2007 (4.1 and 1.6 ng/L, respectively).^8,60^ The decreasing levels of PFOA and PFOS in dust and drinking water could be related to their phase-out in the past two decades.^2^ Among other long-chain PFAAs, PFHxS and PFNA were found at median concentrations of 0.17 ng/L (DF 88%) and 0.11 ng/L (DF 65%), respectively, which were lower than those of the C8 PFAS. The rest of the long-chain PFAAs, were generally detected less frequently than in dust (2.5–49%) and contributed only a minor portion to the total PFAA concentrations (<1%). Perfluoro-4-ethylcyclohexanesulfonic acid (PFECHS), a cyclic C8 PFAA, was detected in 44% of the drinking water samples. Previous studies indicate the PFECHS contamination in drinking water could be related to airports located nearby.^73^ Here, PFECHS was detected more frequently in tap water collected from homes located in a county with a local airport compared to locations without nearby airports (on average, DF 67 vs 37%, respectively).

Among precursors, only a few were detected in 11–31% of drinking water samples, including perfluorooctanesulfonamide (FOSA), perfluorobutanesulfonamide (FBSA), and 6:2 fluorotelomer sulfonate (6:2 FTSA) at concentrations reaching 1 ng/L.

#### Serum

Similar to the dust and drinking water samples analyzed here, ultrashort-chain PFAAs were the most abundant PFAAs in serum as well. Specifically, TFA was the predominant PFAA in serum samples (DF 74%, median of 6.0 ng/mL) and constituted 57% of the serum total PFAA concentration. PFPrA was found in 99% of the samples at a median concentration of 1.0 ng/mL and contributed ∼10% to the serum total PFAA levels. These findings were consistent with those from a recent study from China (sampling year 2017) that reported TFA and PFPrA in serum at median concentrations of 8.5 and 0.48 ng/mL, respectively.^18^ Most of the other short-chain PFAAs were detected in ∼80% of the samples but at lower concentrations and contributed less than 2% to the total PFAA levels.

As expected, PFOA and PFOS were also frequently detected (DF 99% each) in serum. Median concentrations of PFOA and PFOS in serum were 0.63 and 1.5 ng/mL, constituting 5.9 and 14% of the total PFAA concentration, respectively. The concentrations of the C8 PFAS were lower than those of TFA and comparable to those of PFPrA in these samples. PFHxS was found in almost all samples (DF 99%) at levels comparable to those of PFOA and PFOS (median 0.78 ng/mL) and constituted ∼8% of the total PFAA concentration, while the rest of the long-chain PFAAs were only minor contributors (<2%).

Overall, the total ultrashort- and short-chain PFAAs contributed a greater portion to the total PFAA concentration in serum (69%), demonstrating that the levels of the shorter-chain alternatives accumulating in blood exceed those of the legacy long-chain PFAAs. It was recently reported that short-chain PFAAs (*e.g.*, PFBA and PFBS) are able to biomagnify in a terrestrial food chain,^74^ providing a possible explanation for the higher levels of the short-chain PFAAs in blood. Although high solubilities and *K*_ow_ values of the short-chain PFAAs indicate that they would not significantly partition to lipids, multiple studies have demonstrated that C4–C7 PFAAs exhibit strong binding affinities to blood proteins (*e.g.*, human serum albumin, thyroid hormone transport proteins, peroxisome proliferator-activated receptor, and human liver fatty acid binding proteins).^75−78^ Furthermore, TFA was reported to bind to proteinaceous fractions and lipids in biota.^66,75−79^ These findings suggest that the protein binding affinity could be a driving force behind the bioaccumulation mechanism of the ultrashort- and short-chain PFAAs in human blood.

#### Urine

All ultrashort- and short-chain PFAAs were detected in urine with the DF ranging from 1.2 to 88%. PFPeA was the most abundant PFAA in urine (DF 88%; median of 3.2 ng/mL) and constituted 89% of the urine total PFAA concentration. PFBA was the second most abundant PFAA detected in 60% of the urine samples at a median concentration of 0.33 ng/mL and contributed 9.2% to the urine total PFAA concentration. PFPrA was also frequently detected (DF 56%) but at much lower concentrations (median of 0.051 ng/mL) and contributed 1.4% of the urine total PFAA concentration. TFA was detected only in 31% of the samples but was found at high concentrations in some of the samples with its maximum concentration reaching 290 ng/mL. Among the long-chain compounds, PFOA, PFOS, and PFNA were the only compounds detected in urine (DF 7.4–30%). The estimated average renal clearance rates of TFA, PFPrA, and PFBA (7.3, 1.02, and 21 mL/kg/day, respectively), were found to be 1–3 orders of magnitude higher than those of PFOA and PFOS (0.29 and 0.045 mL/kg/day, respectively),^80^ and consequently, the calculated average half-lives of the ultrashort- and short-chain PFAAs (4–62 days) were significantly lower than those of the long-chain PFAAs (646–1533 days, Table S7). The latter findings as well as the higher water solubility of these shorter-chain PFAAs ((0.35–9.7) × 10^5^ mg/L) compared to their longer-chain counterparts (4.7 × 10^–8^–0.21 mg/L) may explain the more frequent detection of the ultrashort- and short-chain PFAAs in urine.^81^

### Concentration Correlations across Matrices

#### PFAAs

The correlations between the logarithmically transformed concentrations of PFAAs detected in more than 50% of the samples across matrices were examined using Spearman correlation analysis, and the results are presented in Table 3. Most of the significant correlations were found between the concentrations of the ultrashort- and short-chain PFAAs in dust and drinking water with the levels in serum. TFA was the only PFAA for which the serum concentrations significantly correlated with both dust and water levels (*r* = 0.40, *p* < 0.001 and *r* = 0.28, *p* = 0.01, respectively). The serum levels of PFBA and PFHpA were significantly correlated with those in water (*r* = 0.23, *p* = 0.04 and *r* = 0.42, *p* < 0.001, respectively). Among the long-chain PFAAs, PFHxS and PFOS levels in dust and serum were significantly and positively associated (*r* = 0.23, *p* = 0.04 and *r* = 0.36, *p* = 0.001, respectively). PFPrA was the only PFAA for which there was a significant association between the levels in water and urine (*r* = 0.24, *p* = 0.03).

**Table 3:** Spearman Correlation Coefficients (*r*) for the Associations among the Natural Log-Transformed PFAA Concentrations in Dust, Drinking Water, Serum, and Urine^a^

	dust–serum	drinking water–serum	dust–urine	drinking water–urine
TFA	0.40*	0.28*		
PFPrA	0.10	–0.07	–0.10	0.24*
PFBA	–0.01	0.23*	0.07	0.02
PFBS	–0.01	0.08		
PFHxA	–0.12	0.14		
PFHpA	0.15	0.42*		
PFHxS	0.23*	–0.02		
PFOA	0.17	0.18		
PFOS	0.36*	–0.04		
PFNA	0.10	0.14		
PFDA	0.15			

a Only PFAAs detected in more than 50% of the samples were included in the analysis. * indicates statistically significant correlations at *p*-value <0.05.

To the best of our knowledge, this is the first report of significant correlations between the concentrations of the ultrashort- and short-chain PFAAs in drinking water and serum samples collected from the general population of the United States. These associations suggest that consumption of drinking water may be a significant exposure pathway for these shorter-chain PFAAs, even in the general population with no known PFAS-contaminated sites nearby. On the contrary, the lack of significant associations between the levels of the long-chain PFAAs, including PFOA and PFOS, in drinking water and serum suggests other sources (*e.g.*, dietary)^82^ for these compounds in this population. Previous research has shown that daily exposure to PFBS, PFPeS, PFHxA, and PFHpA via drinking water intake results in increased levels of these compounds in serum.^30^ Although short-chain PFAAs have shorter half-lives in humans compared to the legacy PFAAs,^30^ these compounds are not efficiently removed in water treatment processes,^10^ which may result in continuous exposure through consumption of tap water. Our findings show that the high abundance of ultrashort- and short-chain PFAAs in drinking water from municipal sources is a potential environmental health problem that should be taken into consideration in assessing the risk of exposure to PFAS in the general population. In addition, a significant positive relationship between the TFA concentrations in dust and those in serum indicates that dust intake could also be an important exposure pathway for this compound.

#### PFAA Precursors

Spearman correlation coefficients of the logarithmically transformed concentrations of PFAA precursors in dust with the concentrations of the ultrashort- and short-chain PFAAs in serum and urine are shown in Table 4. The concentrations of 6:2 PAP, 8:2 PAP, and 6:2/8:2 diPAP in dust were significantly correlated with the serum levels of TFA, PFPrA, PFBA, and PFHpA (*r* = 0.22–0.34, *p* < 0.05). In addition, the dust concentrations of 8:2 diPAP and 8:2 FTOH were significantly correlated with the serum concentrations of PFPrA (*r* = 0.27, *p* = 0.02) and PFHpA in serum (*r* = 0.22, *p* = 0.05), respectively. No significant correlation was found between the dust concentrations of PFAA precursors and urinary PFAA levels.

**Table 4:** Spearman Correlation Coefficients (*r*) for the Associations of the Natural Log-Transformed PFAA Precursor Concentrations in Dust and PFAA Concentrations in Serum and Urine^a^

			serum					urine		
			TFA	PFPrA	PFBA	PFHxA	PFHpA	PFPrA	PFBA	PFPeA
dust	6:2 FTSA	*r*	0.12	0.12	0.09	–0.10	0.07	0.06	0.00	0.10
	8:2 FTSA	*r*	0.12	0.11	0.16	–0.10	0.15	–0.14	0.01	0.10
	6:2 PAP	*r*	0.22*	0.32*	0.20	0.08	0.23*	0.13	–0.01	0.03
	8:2 PAP	*r*	0.30*	0.23*	0.32*	0.05	0.34*	–0.11	0.06	–0.02
	6:2 diPAP	*r*	0.11	0.17	0.02	0.08	0.14	–0.14	–0.10	–0.02
	6:2/8:2 diPAP	*r*	0.23*	0.32*	0.22*	0.10	0.25*	0.13	0.02	–0.01
	8:2 diPAP	*r*	0.15	0.27*	0.12	0.01	0.20	0.15	0.05	0.02
	8:2 FTOH	*r*	0.03	0.01	–0.05	–0.04	0.22*	–0.04	0.04	0.08

a Only analytes detected in more than 50% of the samples were included in the analysis. * indicates statistically significant correlations at *p*-value <0.05.

The strong relationships between the dust concentrations of mono- and diPAPs and the serum concentrations of the ultrashort- and short-chain PFAAs suggest similar sources for these PFAS groups. PAPs are a group of fluorotelomer PFAS that have been widely used in various applications (*e.g.*, food packaging materials, cosmetics, personal care products, and floor finishing).^54,59,83^ PAPs have been found as the most abundant PFAA precursors in indoor dust and on human skin.^54,84^ Previous *in vivo* research shows that 4:2 and 6:2 mono- and diPAPs can transform to the corresponding short-chain PFCAs (4:2 PAP/diPAP to PFBA and PFPeA and 6:2 PAP/diPAP to PFHxA and PFHpA).^51^ Moreover, 6:2 diPAP can break down to produce PFPeA in rat blood.^85^ However, the PFAAs with the shortest chain length, TFA and PFPrA, were not measured in the latter studies. Considering the widespread exposure to PAPs and the evidence of their transformation to short-chain PFAAs in animal and environmental studies, it is possible that these compounds are precursors of short-chain PFAAs in humans. In addition, consumption of fluorinated drugs can also contribute to the burden of TFA in humans.^72^ Further research is warranted to investigate the biotransformation mechanisms of PAPs and other precursors in humans.

### Effect of Housing Characteristics

The total PFAA and PFAA precursor dust levels in homes with carpet (medians 396 and 1070 ng/g, respectively) were 2–4 times higher compared to homes without carpet (168 and 288 ng/g, respectively, *p* < 0.05; Figure S1). In addition, the levels of PFAAs and PFAA precursors in dust collected from homes with less frequent vacuuming (medians 395 and 1200 ng/g, respectively) were up to 3 times higher than in homes that were vacuumed more often (medians 306 and 463 ng/g; *p* = 0.096 and 0.045, respectively; Figure S2). High total PFAA and PFAA precursor concentrations in dust collected from homes with carpet suggest that carpet can be an important source of PFAAs and PFAA precursors in the indoor environment, consistent with previous research on indoor PFAA sources.^53,86^

### Relative Source Contributions (RSC) of Drinking Water and Dust Intake to PFAA Body Burden

The results of the one-compartment toxicokinetic (TK) model are provided in Table S8. Overall, our findings indicate that the median RSC of drinking water intake to the serum total PFAA concentration varied from 1.6 to 19%, which was 2–27 times higher than that of dust intake (medians 0.06–7.0%, ingestion + dermal absorption). The estimated ratios of RSC_water_ to RSC_dust_ for the ultrashort- and short-chain PFAAs were higher (7.1–263) than those for the long-chain PFAAs (0.4–14), suggesting that for the shorter-chain PFAAs consumption of drinking water is a more significant exposure pathway than dust intake. These findings are consistent with previous studies demonstrating a lower significance of PFAA intake from dust ingestion.^87,88^ The RSCs determined here show that dust and water intake only contribute 2.0–20% to the total PFAA body burden, indicating the existence of other exposure pathways for these compounds. For example, the median RSC of drinking water intake was 11% for PFOA, consistent with previously reported findings (0.7–37% of the total PFOA exposure),^89,90^ while for PFOS it was 2.8%. Another potential exposure route could be dietary intake (which was estimated at 66–99% for PFOS exposure in previous studies^91−93^); however, it was not evaluated in this study. In addition, the biotransformation of PFAA precursors frequently detected in food packaging materials,^94^ cosmetics,^58,59^ and indoor dust could also be an indirect pathway for PFAA exposure.^54^

### Limitations

This study had several limitations, including a small sample size and a cohort limited in diversity and geographic coverage. We were not able to determine the contribution of diet or biotransformation of PFAA precursors or fluorinated pharmaceuticals to the total body burden of ultrashort- and short-chain PFAAs. The C2–C5 PFAAs have only one MRM transition and certain chemical interferences may coelute with these compounds because of a shared quantitative ion channel when using low-resolution tandem mass spectrometry for their analysis.^95−97^ Thus, the reported levels of these PFAS should be interpreted with caution. In addition, labeled TFA standards were not available to us when this work started; thus, the extraction efficiency of TFA was not evaluated based on labeled standards. Finally, urinary elimination was assumed to be the primary excretion pathway for the ultrashort- and short-chain PFAAs measured in these samples, and other excretion pathways were not evaluated.

### Implications

This is the first study that reports a substantial prevalence of the ultrashort PFAAs, C2 TFA and C3 PFPrA, in the U.S. indoor environment and the general population. In most of the samples analyzed in this study, the levels of these two ultrashort-chain PFAAs were higher than or comparable to the levels of legacy PFOS and PFOA and constituted on average 66–92% of the total PFAA concentrations. However, the sources of these ultrashort-chain PFAAs in this study remain unknown. The toxicokinetic model applied here shows that consumption of drinking water and dust intake contributed only ∼20% to the total PFAA levels in blood, suggesting other exposure pathways for these compounds. On the other hand, high levels of several PFAA precursors in dust and a strong relationship found between the dust levels of some precursors, such as PAPs, with those of TFA and PFPrA in blood indicate common sources and suggest that biotransformation of PAPs could be a potential indirect source of the ultrashort-chain PFAAs in humans. However, *in vivo* and *in vitro* biotransformation studies of PFAA precursors that have examined the formation of the ultrashort-chain PFAAs are lacking. Our findings warrant urgent research focused on the ultrashort-chain PFAAs to elucidate their sources, potential human exposure pathways, and the effects of these exposures on human health.

## Supporting Information Available

List of target analytes and details of the instrumental methods; MDLs, blank concentrations, surrogate and matrix spike recoveries; toxicokinetic model parameters; and results of the correlations analyses (PDF)
