# Supplementary material for: Elevated Levels of Ultrashort- and Short-Chain Perfluoroalkyl Acids in US Homes and People
Source: Environ Sci Technol. 2023 Oct 11;57(42):15782–93. doi: 10.1021/acs.est.2c06715 (PMC10603771; doi:10.1021/acs.est.2c06715)
Supplement: Supplementary file 1 — es2c06715_si_001.pdf [file es2c06715_si_001.pdf]

SUPPORTING INFORMATION

Elevated Levels of Ultrashort- and Short-Chain Perfluoroalkyl Acids in US Homes and People

Guomao Zheng[1]*, Stephanie M. Eick[2,3], and Amina Salamova[2]*

[1] School of Environmental Science and Engineering,

Southern University of Science and Technology, Shenzhen 518055, China

[2] Gangarosa Department of Environmental Health, Rollins School of Public Health,

Emory University, Atlanta, Georgia 30322, USA

[3] Department of Epidemiology, Rollins School of Public Health,

Emory University, Atlanta, Georgia 30322, USA

*Corresponding authors:

1. Amina Salamova

1518 Clifton Road, NE

Atlanta, Georgia 30322 USA

Email: amina.salamova@emory.edu

2. Guomao Zheng

NO. 1088, Xueyuan Road, Nanshan District,

Shenzhen, Guangdong, China 5180551

Email: zhenggm@sustech.edu.cn

Number of pages: 23

Number of figures: 3

Number of tables: 9

Chemicals and reagents. All native and labeled standards were obtained from Wellington Laboratories, Sigma-Aldrich, and Matrix Scientific (Tables S2 and S3). All individual standards had a purity of ≥ 98%. Oasis weak anion exchange (WAX) solid-phase extraction cartridges (3 cc, 60 mg, 30 µm and 6 cc, 150 mg, 30 µm) were obtained from Waters. Envi-Carb (graphitized non-porous carbon) and centrifugal filters (nylon membrane, 0.2 µm) were purchased from Sigma-Aldrich and VWR International, respectively. Methanol and water were LC/MS grade from Fisher Scientific, along with formic acid, ammonium acetate, acetic acid, and ammonium hydroxide.

Sample analysis. All dust samples were sieved using a 500 µm mesh size sieve and approximately 100 mg of dust was spiked with surrogate standards (Tables S2 and S3) and sonicated in 4 mL of methanol for one hour. The mixture was centrifuged at 3000 rpm for five minutes, the supernatant was transferred to a clean tube, and the extraction was repeated twice with 4 mL of methanol. The supernatants were combined, and the resulting extract was concentrated to 500 µL.

Drinking water samples (300 mL, adjusted to pH 4 using acetic acid) were spiked with surrogate standards and loaded into 50 mL reservoirs coupled with Oasis WAX cartridges (6 cc, 150 mg, 30 µm), which were pre-conditioned with 6 mL of methanol with 1% ammonium hydroxide, followed by 6 mL of methanol and then by 6 mL of water. The cartridges were allowed to dry completely under a vacuum and then eluted with 6 mL of 1% ammonium hydroxide in methanol. The extracts were concentrated to dryness and reconstituted in 200 µL of methanol.

Serum samples (1 mL) fortified with surrogate standards were ultrasonicated in 4 mL of acetonitrile for one hour. The samples were then centrifuged (3000 rpm, five minutes) and the supernatants were transferred to new tubes. Each sample was re-extracted twice (total of three extractions), and the supernatants were combined. The samples were then further concentrated to

~1 mL under a gentle stream of N_2 and diluted with 4 mL of water. The resulting extracts were loaded onto WAX cartridges (3 cc, 60 mg, 30 µm) conditioned with 3 mL of 1% ammonium hydroxide in methanol, followed by 3 mL of methanol and then by 3 mL of water. The column was then washed with 3 mL of water and 3 mL of methanol/water (1:9, v/v) mixture and target analytes were eluted with 3 mL of 1% ammonium hydroxide in methanol. The extracts were evaporated to dryness and reconstituted in 200 µL of methanol.

Urine samples (1 mL) were fortified with surrogate standards, diluted with 1 mL of 1% acetic acid, and then loaded onto WAX cartridges conditioned with 3 mL of 1% ammonium hydroxide in methanol, 3 mL of methanol, and 3 mL of water. The columns were then washed with 3 mL of water and 3 mL of methanol/water (1:9, v/v) and the target analytes were eluted with 3 mL of 1% ammonium hydroxide in methanol. The extracts were concentrated to 1 mL, further cleaned up by adding 100 mg of Envi-Carb, and then vortexed for one minute and centrifuged for five minutes. The supernatant was transferred to a new tube and the residue of Envi-Carb was re-extracted with 1 mL of methanol. The combined supernatants were evaporated to dryness and redissolved in 200 µL of methanol. The urine specific gravity was determined by the refractometer and urinary concentrations were adjusted accordingly. All extracts were filtered through 0.2 µm nylon syringe filters and spiked with a mixture of internal standards (Tables S2 and S3) before instrumental analysis.

Instrumental analysis. Forty-seven per- and polyfluoroalkyl substances (PFAS), including 23 perfluoroalkyl acids or PFAAs (14 perfluoroalkyl carboxylic acids [PFCAs] and 9 perfluorosulfonic acids [PFSAs]), and 24 PFAA precursors (3 fluorotelomer sulfonates [FTSAs], 7 perfluorooctane sulfonamides/perfluorooctane sulfonamidoethanols [FOSAs/FOSEs], 5 polyfluorinated phosphate esters [PAPs], 4 fluorotelomer alcohols [FTOHs], 5 fluorotelomer

acrylates/fluorotelomer methacrylates [FTACs/FTMACs]) were included in this analysis. The complete list of analytes is given in Tables S2 and S3. An ultra-performance liquid chromatograph coupled with a triple-quadrupole mass spectrometer (Agilent 1290 Infinity II UPLC – 6470 QQQ-MS) in the negative electrospray ionization (ESI-) mode was used to analyze PFCAs, PFSAs, FTSAs, FOSAs, and PAPs. Chromatographic separation was achieved using an Acquity UPLC BEH C18 column (50 mm× 2.1 mm, 1.7 µm, Waters) at 40°C. Mobile phases consisted of 2 mM ammonium acetate in water (*A*) and 2 mM ammonium acetate in methanol (*B*). The gradient was 10% *B* for 0.5 min initially, ramped to 40% *B* at 1 min**,** and then increased to 100% *B* at 17.5 min. The instrument was equilibrated for 3.5 min after every run. The injection volume was 5 µL. The separation of trifluoroacetic acid (TFA) and perfluoropropanoic acid (PFPrA) was achieved on a Dionex IonPac AS21 IC column (250mm× 2.0mm, 7 µm, Thermo Fisher Scientific). An isocratic mobile phase, consisting of 80% methanol in water containing 50 mM ammonium acetate (at pH = 9), was used at a flow rate of 0.3 mL/min. The total run time was set to 15 min and the column temperature was 45 °C. The nebulizer, gas flow, gas temperature, capillary voltage, sheath gas temperature, and sheath gas flow for all analytes, including TFA and PFPrA, were set to 25 psi, 10 L/min**,** 300°C, 2800 V, 330°C, and 11 L/min**,** respectively. Data acquisition was conducted in a multiple reaction monitoring (MRM) mode. The optimized MRM transitions, fragmentors, and collision energies for target analytes, surrogate, and internal standards are obtained using the Masshunter Optimizer (Table S2). Although only one pair of transition ions was monitored for TFA (113>69), PFPrA (162.9>119), and PFBA (213>169) due to the low abundance of other product ions, these MRM transitions have been successfully applied to the identification and quantification of these compounds in previous studies.[1-3] To further validate the accuracy of the product ions of PFPrA, the authentic standard of PFPrA was scanned in the tandem mass mode

under different collision energies (5, 10, 20V). *M/z* 119.0 was the most abundant product ion of PFPrA and only a minor portion of *m/z* 69.0 was generated. *M/z* 113.0, considered as the precursor ion of TFA, was not observed as a product ion of PFPrA (Figure S3). These results suggest that the in-source fragmentation of PFPrA is not expected to contribute to the false detection of TFA under the MRM mode applied in the current study. In addition, the chromatographic retention times for TFA and PFPrA were quite different (5.1 min and 4.5 min, respectively) that aided in separation of these two peaks.

FTOHs, FTACs, FTMACs, and FOSEs were analyzed using an Agilent 7890 gas chromatograph (GC) coupled to an Agilent 5975C mass spectrometer (MS) in the electron capture positive ionization (PCI) mode. The injection temperature was set to 200°C and the injection volume was 2 μL. Helium was used as the carrier gas with a flow rate of 1 mL/min. The separation was achieved on a CP-WAX 57 CB capillary column (25 m, 250 μm i.d., and 0.2 μm film thickness, Agilent J&W). The following oven temperature program was used as follows: 60°C kept for 3 min, ramped to 85°C at a rate of 25°C/min, then to 190°C at 3°C/min and kept for 8 min. The MS ion source, quadrupole, and GC/MS transfer line temperatures were maintained at 200°C, 106°C, and 200°C, respectively. The target compounds were analyzed using the selected ion monitoring (SIM) mode. The optimized monitoring fragment ions were selected based on full scan mass spectra (Table S3).

Quality assurance and control. Procedural blank and matrix spike samples were analyzed along with each batch of 12 samples. Procedural blank samples prepared from equivalent volumes of LC/MS grade water (300 mL for water and 1 mL each for serum and urine) and 100 mg anhydrous sodium sulfate for dust were analyzed in each respective sample batch. On average, the levels in blanks constituted less than 20% of the levels in samples. The reported concentrations

were blank corrected by subtracting the average blank levels from sample levels. For compounds detected in blanks, MDLs were set as the average blank levels plus three times the standard deviation of the levels in the procedural blanks. For compounds not detected in blanks, MDLs were calculated using a signal-to-noise ratio of three. Six field blanks were collected using nylon socks (for dust) or empty collection tubes and jars (for water, blood, and urine) briefly opened during sampling. The results for procedural blanks, field blanks, and MDLs are included in Table S4. Quantification of the target analytes and surrogate standards was performed by isotope dilution of the internal standards using calibration curves with concentration ranges of 0.1−500 ng/mL. The list of target analytes and the respective surrogate and internal standards is provided in Table S4. Correlation coefficients in linearity tests were all >0.99, and samples with concentrations exceeding the linearity ranges were diluted to achieve the levels within the concentration ranges of the calibration curves. Identification and quantitation of target analytes were accomplished by Agilent's Mass Hunter Quantitative Analysis Software (version B.08.00), with a retention time tolerance of ± 0.1 min.

Efficiencies of the sample preparation procedures for dust, drinking water, serum and urine samples were assessed by analyzing samples spiked with native compounds of PFAAs and PFAA precursors before the extraction. The spike amounts for each analyte in these samples are provided in Table S5. The absolute recoveries for all analytes in matrix spike samples ranged from 40-145%. Low recoveries were observed for several long-chain PFAAs in urine, including perfluorododecanoic acid (PFDoA), perfluorotridecanoic acid (PFTrDA), perfluorotetradecanoic acid (PFTeDA), and perfluorohexadecanoic acid (PFHxDA) (23-31%), and N-ethylperfluorooctanesulfonamide (EtFOSA) (24-29%) in drinking water, serum, and urine.

Recoveries for surrogate standards ranged from 75 ± 2.4 to 99 ± 1.1% in dust; 50 ± 3.9 to 118 ± 8.9% in water; 79 ± 1.1 to 107 ± 6.3% in serum; and 60 ± 2.2 to 97 ± 3.4% in urine (Table S6).

Data analysis. One-compartment toxicokinetic (TK) models have been successfully applied to assess relative contributions of external sources, such as drinking water, to serum concentrations.[4] We used a TK model to estimate the resulting serum concentrations ($C_{dust\ to\ serum}$ and $C_{water\ to\ serum}$) and relative source contributions (RSCs) of dust (dust ingestion + dust dermal absorption) and drinking water intake ($RSC_{dust\ to\ serum}$ and $RSC_{water\ to\ serum}$) as follows (a steady state for dust and drinking water intake was assumed):

$$C_{dust\ to\ serum} = \frac{(C_{dust} \times Q_{dust} \times F_{uptake} + C_{dust} \times BSA \times DAS \times F_{skin}) \times T_h \times T_{1/2}}{V_D \times BW \times ln(2)} \quad (1)$$

$$C_{water\ to\ serum} = \frac{C_{water} \times DW \times T_{1/2}}{V_D \times ln(2)} \quad (2)$$

$$RSC_{dust\ to\ serum} = \frac{C_{dust\ to\ blood}}{C_{serum}} \times 100\% \quad (3)$$

$$RSC_{water\ to\ serum} = \frac{C_{water\ to\ blood}}{C_{serum}} \times 100\% \quad (4)$$

where C_{water} and C_{dust} are the median concentrations of a PFAA detected in drinking water (ng/L) and dust (ng/g), respectively, $T_{1/2}$ and V_D are the half-life (days) and the volume of distribution (mL/kg) of this PFAA in the human body, DW is the daily drinking water intake (L/day), Q_{dust} is the dust ingestion rate (mg/d), F_{uptake} is the uptake fraction of that PFAA (unitless), BSA is the exposed body surface area (cm^2), DAS is the amount of dust that adhered to skin (mg/cm^2), F_{skin} is the fraction of PFAA absorbed by the skin (unitless), BW is the average body weight (kg), and T_h is the average time spent at home (days). The complete dataset, including $T_{1/2}$

and V_D of individual PFAA and other input parameters applied in the TK model is presented in Table S7.

Due to the limited data on the toxicokinetics of TFA, PFPrA, and PFHxA, their respective $T_{1/2}$ were determined based on an one-compartment model using Equations 5 and 6 adopted from:[5]

$$CL_{renal} = \frac{C_{urine} \times V_{urine}}{C_{serum} \times BW} \quad (5)$$

$$T_{1/2} = \frac{ln(2) \times V_D}{CL_{total}} \quad (6)$$

where C_{urine} and C_{serum} are the median concentrations of a PFAA in paired urine and serum samples (ng/mL). The average daily urine excretion volume (V_{urine}) for females and males is 1.3 L.[5] Because the V_D for TFA, PFPrA, and PFHxA in humans are not available, these V_D values were estimated as 130, 130, and 198 mL/kg, respectively, based on the values available for PFBA and PFHpA.[6, 7] Renal clearance is assumed to be the main pathway of human excretion for PFCAs and PFSAs,[5] thus, CL_{total} was set equal to CL_{renal} (kg/day) in Equation 6. The CL_{renal} values for TFA, PFPrA, and PFBA are given in Table S9.

Table S1. Participant surveys on demographic and housing characteristics.

Thank you for agreeing to take part in our survey, all information that you provide will be kept strictly confidential. This short survey includes a few questions about you, your home and the property (land) on which you live. Please complete the following questions as honestly as possible.

Unless otherwise noted, please provide one answer for each question.

Question	Response
What is your current occupation? *If you have more than one job please list your primary occupation.*	Current Occupation: ________________________________<br>☐ Retired or Not Working<br>☐ Unabled<br>☐ Don't Know/Not Sure<br>☐ Refused
If retired or not working, what was your primary occupation?	Primary Occupation: ________________________________<br>☐ Never Worked<br>☐ Don't Know/Not Sure<br>☐ Refused
How much do you weigh without shoes?	☐☐☐ Pounds (lbs)<br>☐ Don't Know/Not Sure<br>☐ Refused
How tall are you without shoes?	☐☐ feet<br>☐☐ inches<br>☐ Don't Know/Not Sure<br>☐ Refused
During the last 24 hours, how many hours did you spend inside and outside of your home? *Please include both awake and asleep hours* ***for a total of 24 hours combined****.*	☐☐ Hours inside my home *(both awake and asleep)*<br>☐☐ Hours away from my home *(outside, driving, at another location)*<br>☐ Don't Know/Not Sure<br>☐ Refused
Do you vape or smoke cigarettes?	☐ Yes, smokes or vapes inside the house<br>☐ Yes, but never smokes or vapes inside the house<br>☐ No<br>☐ Refused
Does anyone else living in your household currently vapes or smoke cigarettes?	☐ Yes, smokes or vapes inside the house<br>☐ Yes, but never smokes or vapes inside the house<br>☐ No<br>☐ Don't Know/Not Sure<br>☐ Refused

Now we would like to get some information about the house you live in. If you don’t know an exact answer, your best guess is fine.

What type of dwelling do you live in?

- ☐ House
- ☐ Apartment
- ☐ Mobile home
- ☐ Separate living quarters in a facility
- ☐ Other, please specify below

  ____

- ☐ Don’t Know/Not Sure
- ☐ Refused

How many people currently live in your home? *Please include all adults and children.*

- ☐ 1
- ☐ 2
- ☐ 3
- ☐ 4
- ☐ 5
- ☐ More than 5 people in your home
- ☐ Don’t Know/Not Sure
- ☐ Refused

How long have you lived at your current residence?

- ☐ Less than 5 years
- ☐ 5-10 years
- ☐ 11-20 years
- ☐ 21-30 years
- ☐ 31-40 years
- ☐ 41-50 years
- ☐ Over 50 years
- ☐ Don’t Know/Not Sure
- ☐ Refused

Approximately when was your residence built?

- ☐ Built before 1940
- ☐ Between 1940 and 1969
- ☐ Between 1970 and 1999
- ☐ Built after 2000
- ☐ Don’t Know/Not Sure
- ☐ Refused

What types of flooring are present in your home? *Please check all that apply.*

- ☐ Carpet
- ☐ Hardwood
- ☐ Vinyl/linoleum
- ☐ Tile
- ☐ Other (SPECIFY)

  ____

- ☐ Don’t Know/Not Sure
- ☐ Refused

Do any of the rooms in your house have vinyl wallpaper or covering?

- ☐ Yes
- ☐ No
- ☐ Don’t Know/Not Sure
- ☐ Refused

What type of heat do you use in your home? *Check all that apply.*

- ☐ Natural gas
- ☐ Baseboard, heat pump, or other electricity
- ☐ Propane
- ☐ Wood inside the home
- ☐ Coal inside the home
- ☐ Other (including outdoor wood or coal), please specify below

  ____

- ☐ Don’t Know/Not Sure
- ☐ Refused

What is the source of water in your home?

- ☐ Private well
- ☐ County or city water department
- ☐ Cistern/catchment system
- ☐ Pond or stream
- ☐ No water source
- ☐ Don't Know/Not Sure
- ☐ Refused

In the past 30 days, how often did you vacuum your home?

- ☐ Never
- ☐ Some days
- ☐ Most days
- ☐ Everyday
- ☐ Don't Know/Not Sure
- ☐ Refused

What cleaning products do you regularly use in your home? How often do you used them? *Please list the names of the 5 most commonly products used and their frequency of use (daily, weekly, etc.).*

1. __________________________
2. __________________________
3. __________________________
4. __________________________
5. __________________________

- ☐ No cleaning products used
- ☐ Don't Know/Not Sure
- ☐ Refused

Finally, we would like to know a little bit about the land your home is located on. Please respond to the best of your knowledge.

Is your home located on or near a farm?

- ☐ Yes
- ☐ No (SKIP next two questions)
- ☐ Don't Know/Not Sure
- ☐ Refused

What type of farm is your home on or located by?

- ☐ Crops
- ☐ Dairy
- ☐ Cattle
- ☐ Pig
- ☐ Poultry
- ☐ Other livestock

  ____

- ☐ Don't Know/Not Sure
- ☐ Refused

How big, in acres, is the farm near your home?

- ☐ Small (under 500 acres, or <1 sq mile)
- ☐ Medium (between 500-1,000 acres, or between 1 – 1.5 sq miles
- ☐ Large (over 1,000 acres, or 1.5 sq miles and over
- ☐ Don't Know/Not Sure
- ☐ Refused

If you have a garden or are involved in agricultural activities, please list the product names and frequency of use of the 5 most commonly used herbicides/ pesticides/insecticides or other chemical treatments you use in your activities.

1. __________________________
2. __________________________
3. __________________________
4. __________________________
5. __________________________

- ☐ No garden or agricultural activities (SKIP the next question)
- ☐ Don't Know/Not Sure
- ☐ Refused

Do you use biosolids (wastewater irrigation or soil treatment) in your agricultural activities?

- ☐ Yes
- ☐ No
- ☐ Don't Know/Not Sure
- ☐ Refused

Table S2. The optimized MRM transitions, fragmentors, and collision energies for target analytes and surrogate and internal standards analyzed under ESI (-) mode. IS: Internal standard; SS: surrogate standard.

Abbreviation	Compound	Supplier	Precursor ion [M-H/D]-	Fragmentor (volts)	Product ions (m/z)	Collision energy (volts)
TFA	Trifluoroacetic acid	Sigma-Aldrich	113.0	64	69.0	5
PFPrA	Perfluoropropanoic acid	Sigma-Aldrich	162.9	64	119	5
PFBA	Perfluorobutanoic acid	Wellington	213.0	64	169	5
PFPeA	Perfluoropentanoic acid	Wellington	263.0	64	218.9	5
					140.8	5
PFHxA	Perfluorohexanoic acid	Wellington	313.0	73	268.9	5
					119	21
PFHpA	Perfluoroheptanoic acid	Wellington	363.0	78	319	5
					169	17
PFOA	Perfluorooctanoic acid	Wellington	413.1	83	369	5
					169	17
PFNA	Perfluorononanoic acid	Wellington	463.1	83	419	5
					218.9	17
PFDA	Perfluorodecanoic acid	Wellington	513.0	93	468.9	5
					269	17
PFUdA	Perfluoroundecanoic acid	Wellington	563.0	102	518.9	5
					268.9	17
PFDoA	Perfluorododecanoic acid	Wellington	613.0	102	569	9
					269	21
PFTrDA	Perfluorotridecanoic acid	Wellington	663.1	107	619	9
					169	29
PFTeDA	Perfluorotetradecanoic acid	Wellington	713.1	112	668.9	13
					169	29
PFHxDA	Perfluorohexadecanoic acid	Wellington	813.1	121	768.9	13
					168.9	37
PFPrS	Perfluoropropanesulfonic acid	Wellington	249.1	140	80.0	37
		Wellington			98.9	33
PFBS	Perfluorobutanesulfonic acid	Wellington	299.0	149	80.0	37
					98.9	37
PFPeS	Perfluoropentanesulfonic acid	Wellington	349.0	175	80.0	45
					98.9	37
PFHxS	Perfluorohexanesulfonic acid	Wellington	399.0	179	80.0	45
					98.9	41
PFHpS	Perfluoroheptanesulfonic acid	Wellington	449.0	183	80.0	49
					98.9	45

Abbreviation	Compound	Supplier	Precursor ion [M-H/D]-	Fragmentor (volts)	Product ions (m/z)	Collision energy (volts)
PFOS	Perfluorooctanesulfonic acid	Wellington	499.0	208	80.0	101
					98.9	49
PFNS	Perfluorononanesulfonic acid	Wellington	549.0	218	80.0	105
					98.9	49
PFDS	Perfluorodecanesulfonic acid	Wellington	598.9	232	80.0	137
					98.9	53
					98.9	49
					98.9	49
PFECHS	Perfluoro-4-ethylcyclohexanesulfonic acid	Wellington	461.0	98.9	49	29
6:2 PAP	1H,1H,2H,2H-perfluorooctylphosphate	Wellington	443.0	108	97.0	17
					79.0	100
8:2 PAP	1H,1H,2H,2H-perfluorodecylphosphate	Wellington	543.0	108	97.0	17
					79.0	100
6:2 diPAP	Bis(1H,1H,2H,2H-perfluorooctyl)phosphate	Wellington	789.0	132	442.9	17
					97.0	37
6:2/8:2 diPAP	(1H,1H,2H,2H-perfluorooctyl-1H,1H,2H,2H-perfluorodecyl)phosphate	Wellington	889.0	156	443.0	21
					96.9	33
8:2 diPAP	Bis(1H,1H,2H,2H-perfluorodecyl)phosphate	Wellington	989.0	151	542.9	25
					97.0	37
4:2 FTSA	4:2 fluorotelomer sulfonate	Wellington	327.1	136	306.9	21
					81	33
6:2 FTSA	6:2 fluorotelomer sulfonate	Wellington	427.0	164	406.9	25
					81	41
8:2 FTSA	8:2 fluorotelomer sulfonate	Wellington	527.0	179	506.9	29
					81	41
					98.9	29
FBSA	Perfluorobutanesulfonamide	Wellington	298.0	98	78.0	25
					48.1	93
FHxSA	Perfluorohexanesulfonamide	Wellington	397.9	117	78.0	29
					48.1	100
FOSA	Perfluorooctanesulfonamide	Wellington	498.0	169	78.0	37
					48.1	150
MeFOSA	N-methylperfluorooctanesulfonamide	Wellington	512.0	160	169	29
					218.9	25
EtFOSA	N-ethylperfluorooctanesulfonamide	Wellington	526.0	165	169	29
					219	29
					63.0	3
M3PFBA (SS)	Perfluoro[2,3,4-$^{13}C_3$]butanoic acid	Wellington	216.0	64	172	5
MPFHxA (SS)	Perfluoro[1,2-$^{13}C_2$]hexanoic acid	Wellington	315.1	78	270	5
MPFOA (SS)	Perfluoro[1,2,3,4-$^{13}C_4$]octanoic acid	Wellington	417.1	83	372	5

Abbreviation	Compound	Supplier	Precursor ion [M-H/D]-	Fragmentor (volts)	Product ions (m/z)	Collision energy (volts)
MPFUdA (SS)	Perfluoro[1,2-$^{13}C_2$]undecanoic acid	Wellington	565.1	97	520	9
M2PFTeDA (SS)	Perfluoro[1,2-$^{13}C_2$]tetradecanoic acid	Wellington	715.1	116	669.9	13
M3PFBS (SS)	Perfluoro[2,3,4-$^{13}C_3$]butanesulfonic acid	Wellington	302.0	149	80	45
MPFHxS (SS)	Perfluorohexane[$^{18}O_2$]sulfonic acid	Wellington	403.0	169	84	49
MPFOS (SS)	Perfluoro[1,2,3,4-$^{13}C_4$]octanesulfonic acid	Wellington	503.0	198	80	93
M2-8:2 PAP (SS)	1H,1H,2H,2H-[1,2-$^{13}C_2$]perfluorodecylphosphate	Wellington	545.0	113	97.0	17
MPFBA (IS)	Perfluoro[1,2,3,4-$^{13}C_4$]butanoic acid	Wellington	217.0	64	172	5
M8PFOA (IS)	Perfluoro[$^{13}C_8$]octanoic acid	Wellington	421.1	83	376	5
M7PFUdA (IS)	Perfluoro[1,2,3,4,5,6,7-$^{13}C_7$]undecanoic acid	Wellington	570.0	97	525	9
M3PFHxS (IS)	Perfluoro[1,2,3-$^{13}C_3$]hexanesulfonic acid	Wellington	402.0	184	80	45
M8PFOS (IS)	Perfluoro-[$^{13}C_8$]octanesulfonic acid	Wellington	507.0	203	79.9	97
M4-6:2 diPAP (IS)	Bis(1H,1H,2H,2H-[1,2-$^{13}C_2$]perfluorooctyl)phosphate	Wellington	793.0	137	445.0	21

Table S3. The details of the instrumental method for the analysis of analytes using gas chromatographic mass spectrometry in the positive chemical ionization mode.

Abbreviation	Compound Name	Supplier	Quantifier	Qualifier
4:2 FTOH	2-Perfluorobutyl ethanol (4:2)	Wellington	265	227
6:2 FTOH	2-Perfluorohexyl ethanol (6:2)	Wellington	365	327
8:2 FTOH	2-Perfluorooctyl ethanol (8:2)	Wellington	465	427
10:2 FTOH	2-Perfluorodecyl ethanol (10:2)	Wellington	565	527
6:2 FTAcr	1H,1H,2H,2H-perfluorooctyl acrylate	Sigma-Aldrich	419	399
8:2 FTAcr	1H,1H,2H,2H-Perfluorodecyl acrylate	Wellington	519	499
10:2FTAcr	1H,1H,2H,2H-Perfluorododecyl acrylate	Wellington	619	599
6:2 FTMAcr	1H,1H,2H,2H-perfluorooctyl methacrylate	Sigma-Aldrich	433	413
8:2 FTMAcr	1H,1H,2H,2H-heptadecafluorodecyl methacrylate	Matrix Scientific	533	513
MeFOSE	2-(N-methylperfluorooctanesulfonamido)-ethanol	Wellington	558	540
EtFOSE	2-(N-ethylperfluorooctanesulfonamido)-ethanol	Wellington	572	554
M2-8:2 FTOH (SS)	2-Perfluorooctyl-[1,2-$^{13}C_2$]-ethanol(8:2)	Wellington	467	429
M-8:2 FTOH (IS)	2-Perfluorooctyl-[1,1-$^{2}H_2$]-[1,2-$^{13}C_2$]-ethanol(8:2)	Wellington	469	431

Table S4. Target analytes, their respective surrogate and internal standards and mean analyte levels measured in procedural (*n* = 6) and field blanks (*n* = 6) and method detection limits [MDL] for dust (ng/g), drinking water (ng/L), blood (ng/mL), and urine (ng/mL). n.d.: not detected. MDLs were set as the average plus three times the standard deviation of the target analyte levels detected in blanks. For analytes not detected in blanks, a ratio of signal to noise of three was used.

Target analytes	Surrogate standards	Internal standards	Dust, ng/g			Water, ng/L			Serum, ng/mL			Urine, ng/mL		
			Proced. blanks	Field Blanks	MDLs	Proced. blanks	Field blanks	MDLs	Proced. blanks	Field blanks	MDLs	Proced. blanks	Field blanks	MDLs
TFA	M3PFBA	MPFBA	52	40	73	15	12	27	3.4	3.0	4.4	2.4	2.1	3.5
PFPrA	M3PFBA	MPFBA	0.60	0.60	0.69	1.6	1.2	2.8	0.22	0.25	0.28	0.005	0.002	0.04
PFBA	M3PFBA	MPFBA	2.6	2.5	2.7	0.39	0.10	0.49	0.09	0.11	0.11	0.001	n.d.	0.04
PFPeA	M3PFBA	MPFBA	n.d.	n.d.	0.01	n.d.	n.d.	0.01	n.d.	n.d.	0.001	n.d.	n.d.	0.001
PFHxA	MPFHxA	M8PFOA	n.d.	n.d.	0.01	n.d.	n.d.	0.01	n.d.	n.d.	0.002	n.d.	n.d.	0.002
PFHpA	MPFHxA	M8PFOA	n.d.	n.d.	0.02	n.d.	n.d.	0.01	n.d.	n.d.	0.001	n.d.	n.d.	0.001
PFOA	MPFOA	M8PFOA	n.d.	n.d.	0.01	n.d.	n.d.	0.01	n.d.	n.d.	0.003	0.02	0.01	0.03
PFNA	MPFOA	M8PFOA	n.d.	n.d.	0.03	n.d.	n.d.	0.01	n.d.	n.d.	0.001	n.d.	n.d.	0.001
PFDA	MPFOA	M8PFOA	n.d.	n.d.	0.01	n.d.	n.d.	0.02	n.d.	n.d.	0.004	n.d.	n.d.	0.004
PFUdA	MPFUdA	M7PFUdA	n.d.	n.d.	0.02	n.d.	n.d.	0.01	n.d.	n.d.	0.005	n.d.	n.d.	0.005
PFDoA	MPFUdA	M7PFUdA	n.d.	n.d.	0.01	n.d.	n.d.	0.01	n.d.	n.d.	0.002	n.d.	n.d.	0.002
PFTrDA	MPFUdA	M7PFUdA	n.d.	n.d.	0.02	n.d.	n.d.	0.01	n.d.	n.d.	0.003	n.d.	n.d.	0.003
PFTeDA	M2PFTeDA	M7PFUdA	n.d.	n.d.	0.02	n.d.	n.d.	0.01	n.d.	n.d.	0.001	n.d.	n.d.	0.001
PFHxDA	M2PFTeDA	M7PFUdA	0.38	0.23	0.48	n.d.	n.d.	0.02	n.d.	n.d.	0.001	n.d.	n.d.	0.001
PFPrS	M3PFBS	MPFBA	n.d.	n.d.	0.04	n.d.	n.d.	0.01	n.d.	n.d.	0.002	n.d.	n.d.	0.002
PFBS	M3PFBS	MPFBA	n.d.	n.d.	0.03	n.d.	n.d.	0.02	n.d.	n.d.	0.001	n.d.	n.d.	0.001
PFPeS	M3PFBS	MPFBA	n.d.	n.d.	0.01	n.d.	n.d.	0.01	n.d.	n.d.	0.001	n.d.	n.d.	0.001
PFHxS	MPFHxS	M3PFHxS	0.18	0.11	0.28	n.d.	n.d.	0.01	0.01	n.d.	0.02	n.d.	n.d.	0.004
PFHpS	MPFHxS	M3PFHxS	n.d.	n.d.	0.01	n.d.	n.d.	0.01	n.d.	n.d.	0.001	n.d.	n.d.	0.001
PFOS	MPFOS	M8PFOS	n.d.	n.d.	0.02	n.d.	n.d.	0.01	n.d.	n.d.	0.001	n.d.	n.d.	0.001
PFNS	MPFOS	M8PFOS	n.d.	n.d.	0.01	n.d.	n.d.	0.01	n.d.	n.d.	0.003	n.d.	n.d.	0.003
PFDS	MPFOS	M8PFOS	n.d.	n.d.	0.03	n.d.	n.d.	0.01	n.d.	n.d.	0.002	n.d.	n.d.	0.002
4:2 FTSA	MPFOS	M8PFOS	n.d.	n.d.	0.01	n.d.	n.d.	0.02	n.d.	n.d.	0.003	n.d.	n.d.	0.003
6:2 FTSA	MPFOS	M8PFOS	0.35	n.d.	0.45	n.d.	n.d.	0.01	n.d.	n.d.	0.004	n.d.	n.d.	0.004
8:2 FTSA	MPFOS	M8PFOS	n.d.	n.d.	0.01	n.d.	n.d.	0.01	n.d.	n.d.	0.002	n.d.	n.d.	0.002

Target analytes	Surrogate standards	Internal standards	Dust, ng/g			Water, ng/L			Serum, ng/mL			Urine, ng/mL		
			Proced. blanks	Field Blanks	MDLs	Proced. blanks	Field blanks	MDLs	Proced. blanks	Field blanks	MDLs	Proced. blanks	Field blanks	MDLs
6:2 PAP	M2-8:2 PAP	M4-6:2 diPAP	n.d.	n.d.	0.04	n.d.	n.d.	0.01	n.d.	n.d.	0.002	n.d.	n.d.	0.002
8:2 PAP	M2-8:2 PAP	M4-6:2 diPAP	n.d.	n.d.	0.01	n.d.	n.d.	0.04	n.d.	n.d.	0.003	n.d.	n.d.	0.003
6:2 diPAP	M2-8:2 PAP	M4-6:2 diPAP	0.04	0.01	0.14	n.d.	n.d.	0.05	n.d.	n.d.	0.002	n.d.	n.d.	0.002
6:2/8:2 diPAP	M2-8:2 PAP	M4-6:2 diPAP	0.09	0.11	0.19	n.d.	n.d.	0.05	n.d.	n.d.	0.002	n.d.	n.d.	0.002
8:2 diPAP	M2-8:2 PAP	M4-6:2 diPAP	n.d.	n.d.	0.01	n.d.	n.d.	0.01	n.d.	n.d.	0.001	n.d.	n.d.	0.001
EtFOSA	MPFOS	M8PFOS	n.d.	n.d.	0.02	n.d.	n.d.	0.01	n.d.	n.d.	0.002	n.d.	n.d.	0.002
FBSA	MPFOS	M8PFOS	n.d.	n.d.	0.01	n.d.	n.d.	0.02	n.d.	n.d.	0.001	n.d.	n.d.	0.001
FHxSA	MPFOS	M8PFOS	n.d.	n.d.	0.01	n.d.	n.d.	0.01	n.d.	n.d.	0.001	n.d.	n.d.	0.001
MeFOSA	MPFOS	M8PFOS	n.d.	n.d.	0.02	n.d.	n.d.	0.01	n.d.	n.d.	0.001	n.d.	n.d.	0.001
FOSA	MPFOS	M8PFOS	n.d.	n.d.	0.01	n.d.	n.d.	0.01	n.d.	n.d.	0.001	n.d.	n.d.	0.001
PFECHS	MPFOS	M8PFOS	n.d.	n.d.	0.01	n.d.	n.d.	0.02	n.d.	n.d.	0.001	n.d.	n.d.	0.001
4:2 FTOH	M2-8:2 FTOH	M-8:2 FTOH	n.d.	n.d.	0.02	-[a]	-	-	-[a]	-	-	-[a]	-	-
6:2 FTOH	M2-8:2 FTOH	M-8:2 FTOH	n.d.	n.d.	0.03	-[a]	-	-	-[a]	-	-	-[a]	-	-
8:2 FTOH	M2-8:2 FTOH	M-8:2 FTOH	n.d.	n.d.	0.01	-[a]	-	-	-[a]	-	-	-[a]	-	-
10:2 FTOH	M2-8:2 FTOH	M-8:2 FTOH	n.d.	n.d.	0.02	-[a]	-	-	-[a]	-	-	-[a]	-	-
6:2 FTAcr	M2-8:2 FTOH	M-8:2 FTOH	n.d.	n.d.	0.01	-[a]	-	-	-[a]	-	-	-[a]	-	-
8:2 FTAcr	M2-8:2 FTOH	M-8:2 FTOH	n.d.	n.d.	0.01	-[a]	-	-	-[a]	-	-	-[a]	-	-
10:2 FTAcr	M2-8:2 FTOH	M-8:2 FTOH	n.d.	n.d.	0.04	-[a]	-	-	-[a]	-	-	-[a]	-	-
6:2 FTMAcr	M2-8:2 FTOH	M-8:2 FTOH	n.d.	n.d.	0.05	-[a]	-	-	-[a]	-	-	-[a]	-	-
8:2 FTMAcr	M2-8:2 FTOH	M-8:2 FTOH	n.d.	n.d.	0.02	-[a]	-	-	-[a]	-	-	-[a]	-	-
MeFOSE	M2-8:2 FTOH	M-8:2 FTOH	n.d.	n.d.	0.01	-[a]	-	-	-[a]	-	-	-[a]	-	-
EtFOSE	M2-8:2 FTOH	M-8:2 FTOH	n.d.	n.d.	0.01	-[a]	-	-	-[a]	-	-	-[a]	-	-

[a]: These analytes were not measured in water, serum, and urine samples because of their high volatility.

Table S5. Analyte spike amounts (ng) and mean recoveries (%) with their standard errors (SE) in matrix spike samples (n = 10).

	Dust			Water			Serum			Urine		
	Spike amount	Mean	SE	Spike amount	Mean	SE	Spike amount	Mean	SE	Spike amount	Mean	SE
TFA	5	83	3.5	5	68	1.9	5	85	3.6	5	77	3.2
PFPrA	5	86	3.7	5	63	3.9	5	110	4.5	5	64	2.6
PFBA	5	76	3.3	5	92	2.2	5	110	4.5	5	110	4.5
PFPeA	5	82	3.5	5	67	3.3	5	74	3.2	5	97	4.1
PFHxA	5	66	2.8	5	79	5.9	5	98	4.2	5	100	4.4
PFHpA	5	64	2.7	5	89	2.7	5	94	4.0	5	84	3.7
PFOA	5	104	4.4	5	87	3.4	5	90	3.9	5	78	3.3
PFNA	5	97	4.2	5	90	6.7	5	97	4.1	5	66	3.5
PFDA	5	88	3.8	5	53	2.2	5	73	3.1	5	50	2.1
PFUdA	5	95	4.1	5	68	4.5	5	88	3.7	5	51	2.8
PFDoA	5	70	3.0	5	45	9.0	5	53	2.3	5	31	2.0
PFTrDA	5	66	2.8	5	32	5.3	5	66	2.8	5	26	2.4
PFTeDA	5	71	3.0	5	44	8.9	5	63	2.7	5	26	4.2
PFHxDA	5	62	2.6	5	46	5.2	5	61	2.6	5	23	1.0
PFPrS	5	85	3.6	5	130	14	5	110	4.7	5	170	8.2
PFBS	5	76	3.2	5	97	4.6	5	110	4.8	5	150	6.5
PFPeS	5	104	4.4	5	91	4.4	5	110	4.8	5	116	5.1
PFHxS	5	102	4.4	5	88	2.4	5	100	4.3	5	110	4.7
PFHpS	5	125	5.3	5	93	4.4	5	94	4.0	5	150	6.4
PFOS	5	100	4.3	5	92	3.4	5	97	4.2	5	66	3.6
PFNS	5	100	4.3	5	70	4.4	5	84	3.6	5	40	3.0
PFDS	5	82	3.5	5	81	3.8	5	83	3.6	5	38	2.3
4:2 FTSA	5	83	3.5	5	99	12	5	100	4.3	5	150	7.2
6:2 FTSA	5	130	5.5	5	85	12	5	92	3.9	5	120	6.0
8:2 FTSA	5	127	5.4	5	83	14	5	92	3.9	5	98	4.1
6:2 PAP	5	54	2.3	5	72	6.9	5	140	6.1	5	72	5.5
8:2 PAP	5	51	2.2	5	69	3.9	5	130	5.7	5	60	3.7
6:2 diPAP	5	85	3.6	5	45	1.6	5	60	2.6	5	43	1.9
6:2/8:2 diPAP	5	118	5.0	5	51	2.9	5	66	2.8	5	43	1.9
8:2 diPAP	5	92	3.9	5	48	2.5	5	44	1.9	5	40	2.0
EtFOSA	5	58	2.5	5	29	6.1	5	24	1.0	5	25	2.2
FBSA	5	81	3.5	5	80	2.3	5	88	3.8	5	100	4.2
FHxSA	5	81	3.5	5	76	3.0	5	78	3.3	5	97	4.1
MeFOSA	5	75	2.1	5	62	2.1	5	73	2.5	5	69	5.1
FOSA	5	73	3.1	5	81	5.9	5	100	4.3	5	44	1.9
PFECHS	5	96	4.1	5	79	8.9	5	95	4.0	5	91	4.3
4:2 FTOH	20	67	2.9	20	-[a]	-	20	-[a]	-	20	-[a]	-
6:2 FTOH	20	64	2.7	20	-[a]	-	20	-[a]	-	20	-[a]	-
8:2 FTOH	20	72	3.1	20	-[a]	-	20	-[a]	-	20	-[a]	-

	Dust			Water			Serum			Urine		
	Spike amount	Mean	SE	Spike amount	Mean	SE	Spike amount	Mean	SE	Spike amount	Mean	SE
10:2 FTOH	20	70	3.0	20	-[a]	-	20	-[a]	-	20	-[a]	-
6:2 FTAcr	20	56	2.4	20	-[a]	-	20	-[a]	-	20	-[a]	-
8:2 FTAcr	20	62	2.6	20	-[a]	-	20	-[a]	-	20	-[a]	-
10:2 FTAcr	20	73	3.1	20	-[a]	-	20	-[a]	-	20	-[a]	-
6:2 FTMAcr	20	59	2.5	20	-[a]	-	20	-[a]	-	20	-[a]	-
8:2 FTMAcr	20	83	3.5	20	-[a]	-	20	-[a]	-	20	-[a]	-
MeFOSE	20	64	2.7	20	-[a]	-	20	-[a]	-	20	-[a]	-
EtFOSE	20	59	2.5	20	-[a]	-	20	-[a]	-	20	-[a]	-

[a]: These analytes were not measured in water, serum, and urine samples because of their high volatility.

Table S6. Mean surrogate recoveries (with their standard errors [SE]) (%).

	Dust		Water		Serum		Urine	
Surrogate	Mean	SE	Mean	SE	Mean	SE	Mean	SE
M3PFBA	78	2.3	76	1.5	97	1.4	85	3.4
M3PFBS	79	2.4	120	8.9	102	3.4	97	3.4
MPFHxA	77	2.4	78	2.6	90	1.8	72	3.5
MPFHxS	84	2.5	78	2.9	102	1.4	86	3.0
MPFOA	87	2.6	92	0.9	90	1.3	79	2.3
MPFOS	87	2.5	75	1.8	79	1.1	60	2.2
MPFUdA	75	2.4	68	2.0	86	1.4	61	2.3
M2PFTeDA	81	3.6	81	4.4	107	6.3	65	2.7
M2-8:2 PAP	89	1.9	50	3.9	89	1.9	72	3.2
M2-8:2 FTOH	99	1.1	-[a]	-	-[a]	-	-[a]	-

[a]: The surrogate recovery of M2-8:2 FTOH was not measured in water, serum, and urine samples because of its high volatility.

Table S7. Parameters used in the toxicokinetic model to calculate relative source contributions of dust intake and water consumption to the blood total PFAA concentrations.

Parameters		Source
**Volume of distribution (V_D, mL/kg)**		
TFA	130	Estimate from Chang et al. 2008[6]
PFPrA	130	Estimate from Chang et al. 2008[6]
PFBA	130	Chang et al. 2008[6]
PFBS	277	Lau et al. 2020[8]
PFHxA	198	Estimate from Ohmori et al. 2003[7]
PFHpA	198	Ohmori et al. 2003[7]
PFHxS	213	Sundström et al. 2012[9]
PFOA	170	Thompson et al., 2010[10]
PFOS	230	Thompson et al., 2010[10]
PFNA	243	Ohmori et al. 2003[7]
PFDA	394	Ohmori et al. 2003[7]
**Half-lives ($T_{1/2}$, days) (95% confidence level [CI])**		
TFA	12 (7-147)	This study
PFPrA	88 (53-147)	This study
PFBA	4 (3-7)	This study
PFBS	44 (37-55)	Estimate from Xu et al., 2020[11]
PFHxA	62 (NA)	Estimate from Xu et al., 2020[11]
PFHpA	62 (51-80)	Xu et al., 2020[11]
PFHxS	1044 (767-1632)	Xu et al., 2020[11]
PFOA	646 (522-840)	Xu et al., 2020[11]
PFOS	1062 (624-3515)	Xu et al., 2020[11]
PFNA	986 (NA)	Hu et al., 2019[4]
PFDA	1533 (NA)	Zhang et al., 2013[5]
**Water intake parameters**		
Daily water intake (DW, L/day)	2.5	EPA exposure handbook[12]
Body weight (BW, kg)	88	This study
**Dust ingestion**		
Dust intake (Q_{dust}, mg/day)	30	EPA exposure handbook[12]
Uptake fraction (F_{uptake}, unitless)	0.8	Zheng et al., 2020[13]
Time spent at home (T_h, days)	0.67	This study
Body weight (BW, kg)	88	This study
**Dust dermal absorption**		
Exposed body surface area (BSA, cm^2)	4615	Stubbings et al., 2018[14]
Dust amount adhered to skin (DAS, mg/cm^2)	0.01	Stubbings et al., 2018[14]
Contaminant fraction absorbed by skin (F_{skin}, unitless)	0.48	Stubbings et al., 2018[14]
Time spent at home (T_h, days)	0.67	This study
Body weight (BW, kg)	88	This study

NA: not available.

Table S8. Estimated serum concentrations (ng/mL) and relative source contributions (RSC, %) of drinking water and dust (ingestion + dermal absorption) intake to the overall PFAA body burden. Only PFAAs with detection frequencies more than 50% are included.

	Water intake		Dust intake		Undetermined
	Predicted serum levels	RSC	Predicted serum levels	RSC	RSC
TFA	0.299	5.0	0.01	0.17	95
PFPrA	0.191	19	0.009	0.89	80
PFBA	0.003	1.6	0.0002	0.11	98
PFBS	0.008	17	0.00003	0.06	83
PFHxA	0.006	17	0.001	2.0	81
PFHpA	0.002	12	0.0003	1.7	86
PFHxS	0.034	4.4	0.007	0.9	95
PFOA	0.072	11	0.011	1.8	87
PFOS	0.042	2.8	0.023	1.6	96
PFNA	0.018	8.7	0.001	0.6	91
PFDA	0.002	3.1	0.004	7.0	90

Table S9. Estimated renal clearance rates of short-chain PFAAs (mL/kg/day).

Renal clearance rates (95% CI)	
TFA	7.30 (0.61-12.2)
PFPrA	1.02 (0.61-1.71)
PFBA	20.9 (12.5-34.9)
PFPeA	0.18 (0.11-0.30)

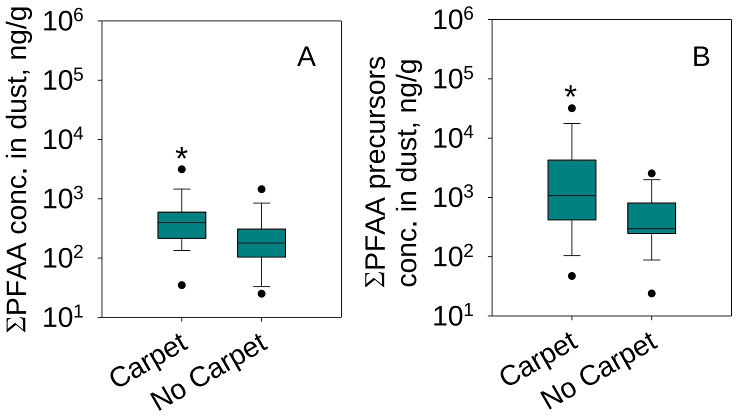

Figure S1. The total PFAA (A) and total PFAA precursor (B) concentrations in dust collected from homes with (n = 66) and without carpet (n = 14). Concentrations are shown as box plots, representing the 25th and 75th percentiles; black lines represent the median, and whiskers represent the 10th and 90th percentiles. The asterisks indicate a statistical difference at $p < 0.05$ based on a Mann−Whitney test.

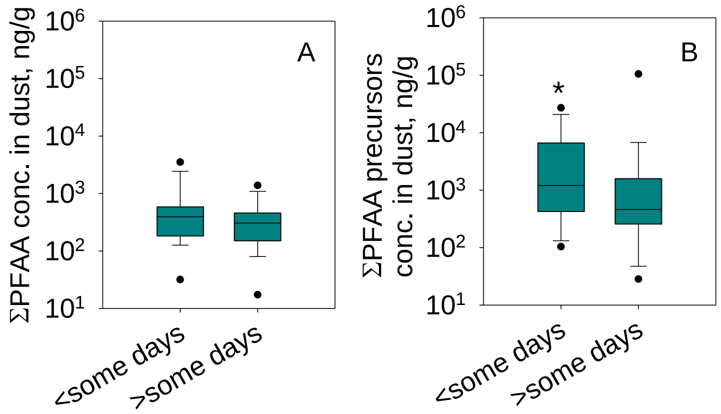

Figure S2. The total PFAA (A) and total PFAA precursor (B) concentrations in dust collected from homes with lower vacuuming frequency (< some days, n = 43) and higher vacuum frequency (> some days, n = 36). Concentrations are shown as box plots, representing the 25th and 75th percentiles; black lines represent the median, and whiskers represent the 10th and 90th percentiles. The asterisks indicate a statistical difference at the $p < 0.05$ level based on a Mann−Whitney test.

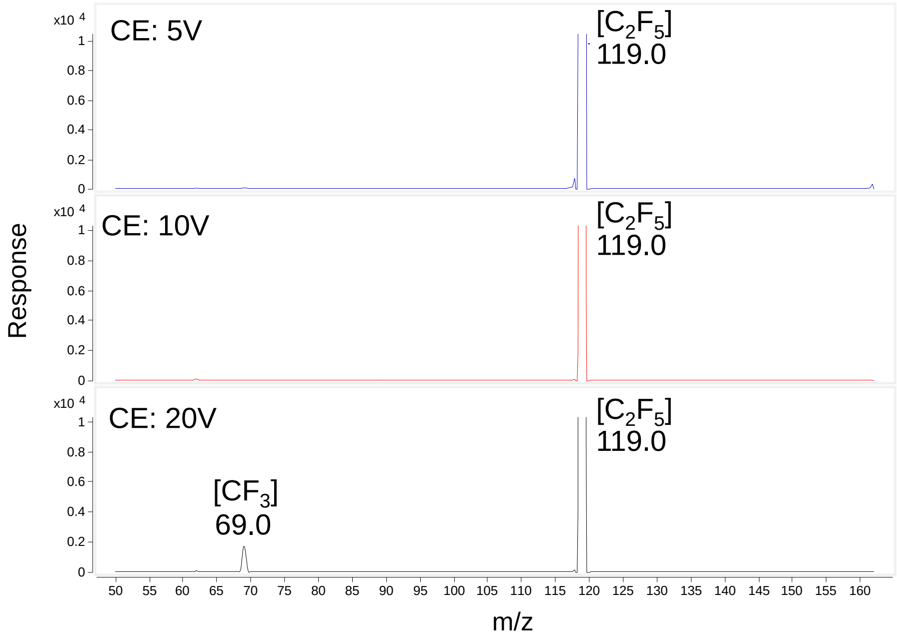

Figure S3. MS/MS spectra of PFPrA under different collision energies.

References

(1) Chen, H.; Zhang, L.; Li, M.; Yao, Y.; Zhao, Z.; Munoz, G.; Sun, H., Per- and polyfluoroalkyl substances (PFASs) in precipitation from mainland China: Contributions of unknown precursors and short-chain (C2-C3) perfluoroalkyl carboxylic acids. *Water Res.* **2019,** *153*, 169-177.

(2) Pickard, H. M.; Criscitiello, A. S.; Persaud, D.; Spencer, C.; Muir, D. C. G.; Lehnherr, I.; Sharp, M. J.; De Silva, A. O.; Young, C. J., Ice core record of persistent short-chain fluorinated alkyl acids: Evidence of the impact from global environmental regulations. *Geophys. Res. Lett.* **2020,** *47*, e2020GL087535.

(3) Wang, B.; Yao, Y.; Chen, H.; Chang, S.; Tian, Y.; Sun, H., Per- and polyfluoroalkyl substances and the contribution of unknown precursors and short-chain (C2–C3) perfluoroalkyl carboxylic acids at solid waste disposal facilities. *Sci. Total Environ.* **2020,** *705*, 135832.

(4) Hu, X. C.; Tokranov Andrea, K.; Liddie, J.; Zhang, X.; Grandjean, P.; Hart Jaime, E.; Laden, F.; Sun, Q.; Yeung Leo, W. Y.; Sunderland Elsie, M., Tap water contributions to plasma concentrations of poly- and perfluoroalkyl substances (PFAS) in a nationwide prospective cohort of U.S. women. *Environ. Health Perspect.* **2019,** *127*, 067006.

(5) Zhang, Y.; Beesoon, S.; Zhu, L.; Martin, J. W., Biomonitoring of perfluoroalkyl acids in human urine and estimates of biological half-life. *Environ. Sci. Technol.* **2013,** *47*, 10619-10627.

(6) Chang, S.-C.; Das, K.; Ehresman, D. J.; Ellefson, M. E.; Gorman, G. S.; Hart, J. A.; Noker, P. E.; Tan, Y.-M.; Lieder, P. H.; Lau, C.; Olsen, G. W.; Butenhoff, J. L., Comparative pharmacokinetics of perfluorobutyrate in rats, mice, monkeys, and humans and relevance to human exposure via drinking water. *Toxicol. Sci.* **2008,** *104*, 40-53.

(7) Ohmori, K.; Kudo, N.; Katayama, K.; Kawashima, Y., Comparison of the toxicokinetics between perfluorocarboxylic acids with different carbon chain length. *Toxicology* **2003,** *184*, 135-140.

(8) Lau, C.; Rumpler, J.; Das, K. P.; Wood, C. R.; Schmid, J. E.; Strynar, M. J.; Wambaugh, J. F., Pharmacokinetic profile of perfluorobutane sulfonate and activation of hepatic nuclear receptor target genes in mice. *Toxicology* **2020,** *441*, 152522.

(9) Sundström, M.; Chang, S.-C.; Noker, P. E.; Gorman, G. S.; Hart, J. A.; Ehresman, D. J.; Bergman, Å.; Butenhoff, J. L., Comparative pharmacokinetics of perfluorohexanesulfonate (PFHxS) in rats, mice, and monkeys. *Reprod. Toxicol.* **2012,** *33*, 441-451.

(10) Thompson, J.; Lorber, M.; Toms, L. L.; Kato, K.; Calafat, A. M.; Mueller, J. F., Use of simple pharmacokinetic modeling to characterize exposure of Australians to perfluorooctanoic acid and perfluorooctane sulfonic acid. *Environ. Int.* **2010,** *36*, 390-397.

(11) Xu, Y. Y.; Fletcher, T.; Pineda, D.; Lindh, C. H.; Nilsson, C.; Glynn, A.; Vogs, C.; Norstrom, K.; Lilja, K.; Jakobsson, K.; Li, Y., Serum half-Lives for short- and long-chain perfluoroalkyl acids after ceasing exposure from drinking water contaminated by firefighting foam. *Environ. Health Perspect.* **2020,** *128*, 077004.

(12) EPA, U. S., Exposure factors handbook. *Edition (Final) (Washington, DC)* **2011**.

(13) Zheng, G.; Schreder, E.; Boor, B.; Salamova, A., Indoor exposure to per- and polyfluoroalkyl substances (PFAS) in the childcare environment. *Environ. Pollut.* **2020,** *258*, 113714-113714.

(14) Stubbings, W. A.; Schreder, E. D.; Thomas, M. B.; Romanak, K.; Venier, M.; Salamova, A., Exposure to brominated and organophosphate ester flame retardants in U.S. childcare environments: Effect of removal of flame-retarded nap mats on indoor levels. *Environ. Pollut.* **2018,** *238*, 1056-1068.
